# Polypharmacy and its association with dementia, Parkinson’s disease, and mortality risk in UK adults: a multistate modeling approach

**DOI:** 10.1007/s11357-025-01586-w

**Published:** 2025-03-13

**Authors:** Jordan Weiss, May A. Beydoun, Michael F. Georgescu, Ana I. Maldonado, Hind A. Beydoun, Nicole Noren Hooten, Jack Tsai, Minkyo Song, Allen Nieva, Michele K. Evans, Alan B. Zonderman

**Affiliations:** 1 Optimal Aging Institute, New York University Grossman School of Medicine, New York, NY USA; 2 Division of Precision Medicine, Department of Medicine, New York University Grossman School of Medicine, New York, NY USA; 3 Laboratory of Epidemiology and Population Sciences, National Institute on Aging, NIA/NIH/IRP, 251 Bayview Blvd, Suite 100, Baltimore, MD 21224 USA; 4 U. S. Department of Veterans Affairs, Palo Alto, CA 94304 USA; 5 U.S. Department of Veterans Affairs, VA National Center on Homelessness Among Veterans, Washington, DC 20420 USA; 6 Department of Management, Policy, and Community Health, School of Public Health, University of Texas Health Science Center at Houston, Houston, TX 77030 USA; 7 Department of Psychology, Princeton University, Princeton, NJ 08544 USA

**Keywords:** Polypharmacy, Dementia, Parkinson’s disease, All-cause mortality, Multistate models, Aging

## Abstract

Polypharmacy is common among older adults and has been linked to adverse outcomes such as dementia, Parkinson’s disease (PD), and mortality. However, its influence on transitions between these health states remains understudied in large, population-based cohorts. Using data from 361,970 UK Biobank participants aged 50 and older with up to 15 years of follow-up, we examined the association between polypharmacy, defined as the use of five or more medications, and transitions between health states: healthy, dementia, PD, and mortality. Multistate parametric models, including Weibull regression, were employed to estimate these associations, adjusting for demographics, socioeconomic status, cardiovascular health, and comorbidities. Latent class analysis was used to identify specific medication combinations associated with health transitions. Polypharmacy was significantly associated with higher risks of transitioning from healthy to dementia (hazard ratio [HR], 1.15; 95% CI, 1.07–1.23) and from healthy to death (HR, 1.11; 95% CI, 1.08–1.09). Women exhibited better cardiovascular health but higher polypharmacy prevalence compared to men. Latent class analysis revealed that certain medication combinations, such as omega-3 fatty acids and multivitamins, were inversely associated with dementia and mortality, independent of polypharmacy status. These findings highlight the complex relationship between polypharmacy and health transitions in older adults. Careful medication management may mitigate risks associated with polypharmacy, particularly among individuals at risk for neurodegenerative diseases. Further research is warranted to investigate the potential protective effects of specific medication combinations on health outcomes.

**Supplementary Information:**

The online version contains supplementary material available at 10.1007/s11357-025-01586-w.

## Introduction

The concurrent use of multiple medications by a patient, known as polypharmacy (POLYPH), is becoming more widespread among older adults (1, 2). POLYPH has been associated with a variety of health outcomes (3, 4) and can exacerbate disease progression and complicate treatment, particularly in the context of dementia and Parkinson’s disease (PD) (4–20). Dementia, characterized by significant cognitive impairment affecting daily activities, presents a major global public health challenge (21, 22). POLYPH has been associated with cognitive decline, potentially contributing to adverse cognitive outcomes through drug interactions, side effects, and increased medication burden (4, 5). Older adults, often managing multiple conditions, are particularly susceptible to these cognitive effects due to frequent medication use (23). Polypharmacy has also been linked to PD, a degenerative neurological illness primarily affecting mobility (19). Managing PD often requires complex medication regimens, increasing the risk of polypharmacy and its associated dangers (16), underscoring the importance of precise drug management and regular treatment plan evaluations for PD patients to mitigate these risks. Moreover, in addition to influencing the incidence of neurodegenerative diseases, POLYPH also affects mortality rates (24–29, 4) primarily due to drug interactions and increased adverse effects (12, 13). Thus, understanding the relationship between POLYPH, neurodegenerative illnesses, and mortality is critical in developing strategies to improve medication management and the well-being of older adults.

Prior research has primarily focused on the effects of POLYPH on specific health outcomes. Few studies have examined how POLYPH affects the transitions between states of health, including death. Furthermore, the impact of POLYPH on these transitions remains unknown, despite common characteristics that may skew the results, such as socioeconomic status and cardiovascular health risk factors. To fill this gap, the current study employed a large sample from the UK Biobank to evaluate the relationship between POLYPH and changes in four health conditions: “healthy,” “PD,” “dementia,” and “death.” The study also looks at gender differences in these relationships, providing a comprehensive understanding of the impact of POLYPH on neurodegenerative diseases and death.

## Methods

### Database

The UK Biobank is a large, prospective cohort study established between 2006 and 2010, enrolling slightly over 500,000 participants aged 37–73 residing in the UK (details are presented elsewhere (30)). Participants completed self-administered questionnaires and in-person interviews across 22 geographically distributed assessment centers in England, Scotland, and Wales (30). The study collected biological samples and extensive phenotypic data (30). Ethical approval for the UK Biobank was granted by the Northwest Multi-Centre Research Ethics Committee. The current project (77963) has received the necessary approvals from both the UK Biobank and the National Institutes of Health Institutional Review Board.

### Mortality linkage

Death registry was used for linkage of the entire UK Biobank cohort as outlined elsewhere: https://biobank.ndph.ox.ac.uk/ukb/ukb/docs/DeathLinkage.pdf. The present study utilized only the date of death provided by the UK Biobank dataset, even though the latter also provided primary as well as contributory causes of death, through the ICD-10 coding system.

### Dementia outcome

The study used algorithmically generated data from hospital registry linkages, specifically fields 42018 and 42020, to analyze dementia cases. Further details on this methodology are available at (https://biobank.ndph.ox.ac.uk/ukb/ukb/docs/alg_outcome_main.pdf). Participants whose dementia onset age preceded their baseline assessment age were excluded from the analysis (https://biobank.ndph.ox.ac.uk/ukb/ukb/docs/alg_outcome_main.pdf). We identified incident Alzheimer’s disease (AD) cases using ICD-10 codes F00 and G30, while other dementia types, including vascular dementia (F01, I67.3), were classified using additional codes (A81.0, F00, F01, F02, F03, F05, G30, G31.0, G31.1, G31.83, I67.3). The method described previously was employed to determine the date of the first occurrence of any type of dementia among many outcomes of interest (https://biobank.ndph.ox.ac.uk/ukb/ukb/docs/alg_outcome_main.pdf).

### PD

PD was identified using an algorithm similar to that which we used for dementia, with the ICD-10 code G20 used to denote PD. This identification method combined hospital records, death records, and self-reports. A validation study of 20,000 UK Biobank participants demonstrated a positive predictive value of 91% for this approach (https://biobank.ndph.ox.ac.uk/ukb/ukb/docs/alg_outcome_main.pdf).

### Polypharmacy

The main exposure of interest, namely POLYPH, was defined using UKB field 137. This variable was then categorized as POLYPH^−^ or 0–1 medications used and POLYPH^2+^ or 2^+^ medications used. In part of the analysis with multistate modeling, POLYPH was coded as 0–4 medication used (POLYPH^−^) vs. 5+ medications used (POLYPH^5+^) to assess the effect of moving the threshold to a higher level as suggested in a recent systematic review (26), and more details on the concept are provided by a technical World Health Organization report (https://www.who.int/docs/default-source/patient-safety/who-uhc-sds-2019-11-eng.pdf). In fact, the recent systematic review describes all cutoffs used and states that 5+ is the most common one, which we used in conjunction with 2+ cutoff (26).

### Commonly used medications

To further investigate which medication groups contributed to the association between exposure and outcome, we identified medications used by at least 5000 participants (48 out of 6745 total medications listed) from the UK Biobank cohort (total *N* = 500,000), as recorded in UKB field 20003 at instance 0 (baseline assessment), covering an array of 48 possible specific medications. These data were used to conduct a latent class analysis (LCA) and generate probabilities of membership in each of these latent classes, as well as a predicted class membership, as detailed in Supplementary methods 2.

### Covariates

We adjusted for the following potential demographic confounders including sex, baseline age, race/ethnicity (White, Black, South Asian, and Others), and household size. Socioeconomic characteristics were also accounted for, given their putative associations with both the outcomes of interest and the main exposure. Those included educational attainment, household income, and occupation. Educational attainment was re-coded as low (“CSEs/Equivalent,” “NVQ/HND/HNC/Equivalent,” or “Other professional qual”), intermediate (“O Levels/GCSEs/Equivalent” or “A/AS Levels Equivalent”), or higher level (“College/University”). Total household income before tax was divided into five categories (“£18,000,” “£18,000–£29,999,” “£30,000–51,999,” “£52,000–£100,000,” “>£100,000”). Occupation (UKB field 20277) was assessed at baseline assessment using the SOC2000 coding system, a hierarchical structure that assigns categories with integers. We grouped these occupations into broader categories in descending order of SES ranging from 1 “managers and senior officials” to 9 “elementary occupations” (categories detailed in Table 1).

**Table 1:** Study sample characteristics by sex: UK Biobank 2006–2021

	Overall (*N*=361,970)	Men (*N*=167,376)	Women (*N*=194,594)	*P*_sex_
Demographic				
Baseline age, y, mean±SE	60.42±0.01	60.73±0.01	60.16±0.01	<0.001
Sex, % female	53.7	--	--	n/a
Race/ethnicity				
White	95.8	95.8	95.8	(Ref)
Black	1.1	1.0	1.0	<0.001
South Asian	1.5	1.8	1.8	<0.001
Other	1.6	1.5	1.5	<0.001
Non-White, %	4.2	4.2	4.2	0.70
Household size	2.234±0.002	2.311±0.003	2.168±0.003	<0.001
Socioeconomic				
Townsend Deprivation Index	−1.507±0.005	−1.490±0.007	−1.522±0.007	0.001
*Education*				
Low	21.7	24.0	19.8	<0.001
Intermediate	39.5	34.7	43.5	(Ref)
High	38.8	41.3	36.6	<0.001
*Income*				
Less than £18,000	25.8	23.2	28.2	<0.001
£18,000–£29,999	27.8	26.7	28.9	(Ref)
£30,000–£51,999	24.7	25.6	23.8	<0.001
£52,000–£100,000	17.3	19.3	15.4	<0.001
Greater than £100,000	4.4	5.2	3.7	<0.001
*Occupation*				
Managers and senior officials	16.1	21.4	11.3	<0.001
Professional occupation	24.1	25.7	22.6	(Ref)
Associate professional/technical occupation	16.2	14.2	18.0	0.001
Administrative/secretarial occupations	16.4	6.5	25.5	<0.001
Skilled trades occupations	7.6	13.9	1.7	<0.001
Personal service occupations	6.0	2.2	9.5	<0.001
Sales/customer service occupations	3.7	1.8	5.4	<0.001
Process plants/machine operatives	4.7	8.6	1.2	<0.001
Elementary occupations	5.2	5.6	4.8	0.35
SES	−0.0496±0.0012	−0.0300±0.0018	−0.066±0.002	<0.001
SES_res_	−6.06e-12±0.0009	+0.0222±0.0014	−0.019±0.001	<0.001
Life’s essential 8, mean±SE				
Total score	497.8±0.2	485.5±0.23	508.38±0.22	<0.001
LE8z_rev	−6.22e-10±0.002	+0.098±0.002	−0.077±0.002	<0.001
Lifestyle score	255.00±0.11	248.79±0.16	260.34±0.14	<0.001
Biological score	242.51±0.11	236.45±0.15	247.7±0.16	<0.001
Co-morbidity index	2.116±0.003	2.072±0.005	2.153±0.005	<0.001
Self-rated health				
Excellent	16.2	15.6	16.8	0.29
Good	58.7	56.7	60.5	(Ref)
Fair	20.8	22.7	19.1	<0.001
Poor	4.2	5.0	3.6	<0.001
Polypharmacy, 2+ medications, % yes	57.4	55.3	59.2	<0.001
Polypharmacy, 5+ medications, % yes	20.6	20.5	20.7	0.042
Cumulative incidence, %				
All-cause dementia	1.7	2.0	1.5	<0.001
Parkinson’s Disease	0.7	1.0	0.5	<0.001
All-cause mortality, %	8.7	11.3	6.4	<0.001

Abbreviations: AD, Alzheimer’s disease; LE8, Life’s essential 8; PRS, polygenic risk score; Ref, referent category; SE, standard error; UK, United Kingdom

Notes: No multiple imputation was carried out in this analysis. P-value is associated with the parameter for sex in bivariate linear and multinomial logistic regression analyses, with the main outcome being a continuous or categorical characteristic, respectively. (Ref) is the referent category in the multinomial logistic regression model. Values are means±SE or percentages. Note that sample sizes vary across LE8 sub-scores and components as well as SES components due to proration

The Townsend Deprivation Index (TDI) was estimated using national census statistics, accounting for car ownership, home overcrowding, owner occupation, and unemployment. Higher TDI scores indicate greater socioeconomic deprivation. The index was computed immediately before participants joined the UK Biobank and is based on the preceding national census output areas, assigning each participant a score corresponding to the output area of their postcode.

We standardized all SES factors and calculated them such that higher scores reflected higher SES. An overall SES z-score was calculated using proration, requiring at least two of the four measures to calculate an average z-score. An alternative SES z-score (SES_res_), independent of TDI, was derived by regressing the overall SES z-score on TDI, with residuals representing education, income, and occupation variability.

We also included markers of cardiovascular health (CVH) using the American Heart Association’s “Life’s Essential 8” (LE8). The LE8 score, which ranges from 0 to 800, includes sleep health in addition to the original seven factors from Life’s Simple 7 (LS7): diet quality, increased physical activity, reduced cigarette smoking, lower BMI, total cholesterol, fasting blood glucose levels, and optimal blood pressure. The LE8 score was standardized and reverse-coded, with higher scores reflecting poorer CVH. Finally, we accounted for the number of comorbid conditions and self-rated health. We used two data fields (134 and 135) to create indices for cancer and non-cancer comorbidities at the baseline assessment. Self-rated health (excellent, good, fair, poor) was also assessed at baseline, with higher scorings indicating poorer health.

### Study sample selection

The initial UK Biobank sample, excluding those withdrawing consent, consisted of 502,160 individuals, with 384,486 of them being 50 years old or older at the initial recruitment. The dataset included information on 362,937 individuals’ socio-demographic, socioeconomic status (SES), lifestyle, and biological traits, which were necessary for analyzing all the key outcomes, exposures, and variables. After excluding 967 cases of pre-existing PD or dementia, the final sample size comprised 361,970 people who were free of dementia and PD. The participants were followed for up to 15 years for all three outcomes of interest (Figure S1).

### Statistical methods

The analyses were conducted using Stata 18.0 software (31). We first computed descriptive statistics, using means and proportions to characterize our sample overall and by sex. We then used time-to-event analysis to examine the associations between exposure and outcome, with time on study used as the underlying time metric. The study exit time was defined as either the age at which an incident or absorbing event occurred (such as all-cause dementia, PD, or all-cause mortality) or the age at which censoring occurred (either death or the end of follow-up, which was set to October 31, 2021, in the case of dementia and PD). In order to assess variations in the likelihood of event-free survival, we calculated Kaplan-Meier survival rates at different follow-up ages. We then compared these rates using a log-rank test across the two exposure groups (“2+”=1 vs. “0–1”=0 for medications used, or the POLYPH^2+^ exposure). Afterwards, Cox proportional hazards (PH) models were used to examine the association between POLYPH^2+^ and each incident event of interest, namely PD, all-cause dementia, and all-cause mortality. These models were adjusted for age at baseline, sex, racial minority status (non-White vs. White), number of individuals living in the same household, the TDI z-score reflecting area-level socio-economic deprivation, SES_res_ z-score reflecting individual-level socio-economic status, and LE8_zrev_ reflecting poorer CVH with a higher score, the number of co-morbidities, and self-rated health. We assessed the validity of the primary Cox proportional hazards models by examining Schoenfeld residuals and employing additional techniques to identify any potential violations of the assumption of proportional hazards. For this portion of the analysis, we stratified our sample by sex and examined the differences between males and females by including a two-way interaction between sex and POLPH^+^ exposure in the fully adjusted Cox proportional hazards model. A sensitivity analysis was performed using the Royston-Parmar flexible parametric model, incorporating limited cubic splines to represent baseline age. The model had 3 degrees of freedom and an interaction term with baseline age (32).

We extended our analysis by modeling transitions between health states and mortality using multistate models, which provided deeper insights into the relationships between variables and health transitions (33). While semiparametric strategies, such as the Cox model, are widely used, the benefits of a fully parametric approach are not as well acknowledged (33). Parametric approaches offer several advantages, including the ability to predict, extrapolate, and quantify data. These benefits are especially valuable when used in conjunction with customized treatment and substantial registry-based data sources (33). In our current investigation, we employed a systematic method to evaluate the suitability of the Weibull model compared to other parametric options. After demonstrating that the Weibull regression model produced similar outcomes to the most suitable model, our final objective was to extrapolate the variations in survival between different exposure groups from this model. This portion of the analysis consisted of procedures that are detailed in Supplementary methods 1.

To summarize these steps, we analyzed the association between exposure to certain medications and health outcomes in individuals with PD or dementia. The dataset was arranged using Stata’s *msset* command, incorporating three events of interest and their corresponding age at occurrence. The data was organized into four states: “healthy,” “PD,” “dementia,” and “deaths.” A transition transformation matrix was created using the *msboxes* command, and the Aalen-Johansen estimates of transition probabilities were calculated. Parametric survival models were estimated using POLYPH as the primary variable of interest, considering factors such as age at baseline, sex, race, household size, TDI z-score, SES_res_, LE8_z_rev_, the co-morbidity index, and self-rated health. POLYPH^2+^ and POLYPH^5+^ were defined as both 2+ and 5+ medications, respectively, in this part of the analysis. Survival probabilities were estimated for each transition and associated model, using Kernel-weighted local polynomial smoothing throughout the age of follow-up, assessing differences in survival probability between exposed and unexposed groups. A sensitivity analysis was performed to compare the fully adjusted parametric model with different distributional specifications. The UK Biobank field 20003 was used to examine which groups of medications explained any association between exposure and outcome. Latent class analysis (LCA) was conducted to generate probabilities of membership in each latent class, as well as a predicted discrete class membership variable. We used improvements in the Akaike Information Criterion (AIC) and the Bayesian Information Criterion (BIC) to select class sizes. A mediation analysis was conducted using generalized structural equations (GSEM) models to explain detected associations between each latent class and the outcome of interest through POLYPH^2+^ or POLYPH^5+^(2^+^ and 5^+^ thresholds). The direct effect of each latent class of medications was of primary interest in these models. Latent classes were labeled and visualized as heat maps, based on a series of bivariate logistic regression models producing Log_e_(odds ratio) of each latent class as modeled against each of the common medications from which the latent classes were estimated.

## Results

The study sample comprised 361,970 individuals from the UK Biobank who were 50 years of age or older. Sex differences were identified in characteristics including age, race, household size, SES indices, and LE8 total and sub-scores (Table 1). Compared to men, women demonstrated elevated scores in LE8 total, lifestyle, and biological sub-scores, suggesting better cardiovascular health (CVH) and self-rated health. However, women exhibited lower socio-economic position as measured by TDI, educational achievement, income, occupation, as well as SES z-score that combined all four metrics and SES_res_, a higher mean co-morbidity index and a greater likelihood of POLYPH, using both the 2+ and 5+ threshold definitions.

**Fig. 1:**
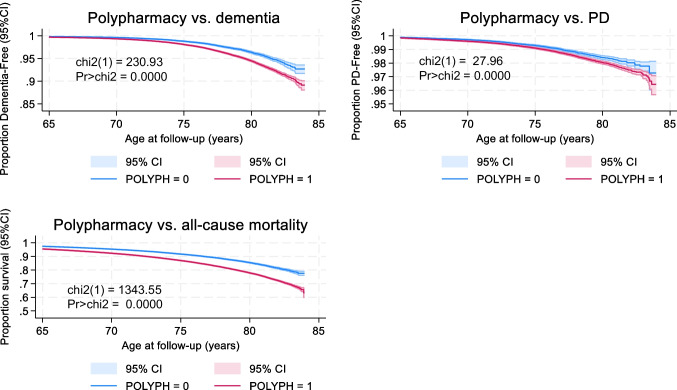
Polypharmacy (2+ medications) by each of 3 outcomes: all-cause dementia, PD, and mortality: UK Biobank 2006–2021. *Abbreviations*: PD, Parkinson’s disease; POLYPH, polypharmacy; UK, United Kingdom. *Notes:* Kaplan-Meier estimates of probabilities in all-cause dementia-free, PD-free, and survival states

The Kaplan-Meier curves demonstrate a strong correlation between POLYPH (2+ definition) and a higher likelihood of developing all-cause dementia, PD, and all-cause mortality (Fig. 1, *P*<0.001, log-rank test). The average duration of observation for event-free survival was approximately 12–13 years. The observation period started from the baseline assessment years of 2006–2010 and continued until October 31, 2021. The study was designed to have a follow-up period of up to 15 years when data was made available for our present analysis. During this time frame, the rate of occurrence was 1.4 instances of all-cause dementia per 1000 person-years, 0.58 cases of incident PD per 1000 person-years, and 7.1 deaths per 1000 person-years. In terms of cumulative incidence and all-cause mortality, all three outcomes occurred at a higher relative frequency among men compared to women, throughout the follow-up time. Throughout the follow-up period, starting from the initial assessment age, there were a total of 31,367 deaths within the selected sample of 361,970 individuals. Younger persons and women had a higher probability of being selected as part of the final sample in a multivariable-adjusted logistic regression model. The major variables in the model were age, sex, and race (non-White vs. White). The outcome variable was the selection status.

Table 2 summarizes the relationships of POLYPH^2+^ with the occurrence of dementia, PD, and all-cause mortality. These associations were examined using Cox proportional hazards models that were adjusted for age, sex, racial minority status, household size, TDI z-score, SES_res_, LE8_z_rev_, the co-morbidity index, and self-rated health. The analysis was conducted both before and after stratification by sex. POLYPH was found to be linked with the occurrence of all-cause dementia in males and females combined (HR=1.15, 95% CI: 1.08, 1.22, *P*<0.001). There was no noticeable variation based on gender. PD exhibited an overall hazard ratio (HR) of 1.07, with a 95% confidence interval (CI) of 0.98 to 1.18 (*P*=0.13). POLYPH was significantly linked with all-cause mortality (HR=1.14, 95% CI: 1.11–1.17, *P*<0.001). There was a notable difference between men and women, with a stronger association observed among males (HR=1.16 in men vs. HR=1.10 in women, *P*<0.001).

**Table 2:** Association between polypharmacy and each of 3 types of events, overall and by sex: Cox PH models^a^, UK Biobank 2006–2021

	Polypharmacy, 2+ medications vs. ≤1 HR with 95% CI	P_polyph_
*All-cause dementia*		
Overall, *N*=361,970	1.15 (1.08;1.22)	<0.001
Men, *N*=167,376	1.18 (1.08;1.29)	<0.001
Women, *N*=194,594	1.11 (1.01.;1.22)	<0.001
*Parkinson’s disease*		
Overall, *N*=361,970	1.07 (0.98;1.18)	0.13
Men, *N*=167,376	1.01 (0.90;1.14)	0.84
Women, *N*=194,594	1.18 (1.01;1.39)	0.033
*All-cause mortality*		
Overall, *N*=361,970	1.14 (1.11;1.17)	<0.001
Men, *N=*167,376	1.16^b^ (1.12;1.20)	<0.001
Women, *N*=194,594	1.10^b^ (1.05;1.15)	<0.001

*Abbreviations*: *CI*, confidence interval; *HR*, hazard ratio; *LE8*, Life’s Essential 8; *PD*, Parkinson’s disease; *PH*, proportional hazards; *SES*, socio-economic status z-score; *SES*_*res*_, residual socio-economic status (z-score) from linear model of SES → TDI; *TDI*, Townsend Deprivation Index; *UK*, United Kingdom

^a^All Cox proportional hazards models were adjusted for baseline age, sex, race/ethnicity (non-White vs. White), household size, LE8_zrev_ TDI z-score, SES residual z-score, co-morbidity index, and self-rated health. Interaction between polypharmacy and sex was tested, by including a 2-way interaction term in the main adjusted model. Values are hazard ratios with 95% CI

^b^*P*<0.05 for null hypothesis that γ = 0, whereby γ is the parameter for 2-way interaction between sex and polypharmacy in the unstratified fully adjusted model

**Fig. 2:**
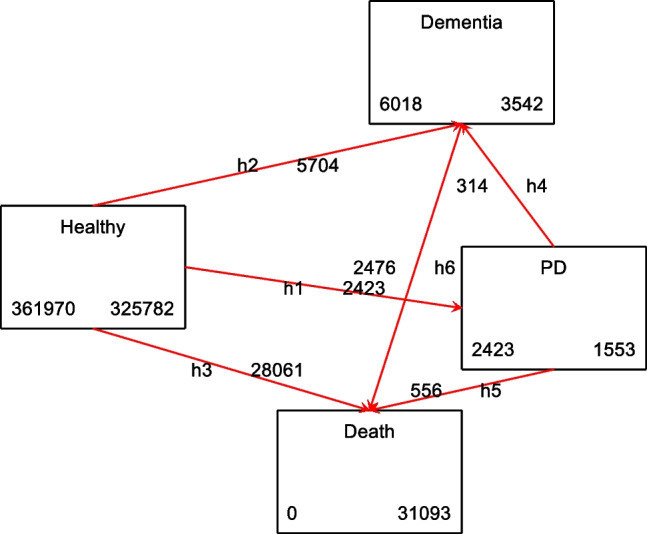
Multistate transition boxes: UK Biobank 2006–2021. *Abbreviations*: PD, Parkinson’s disease. *Notes:* Multistate boxes obtained from the *msboxes* command after *msset* in Stata, produces number of participants in each state, those that leave that particular state to transition into a different state and the remaining participants in that state. The sample sizes shown are within the final selected sample (see Figure S1)

A multistate analysis was conducted, resulting in the identification of 6 health state transitions by specifying events and event timings for 3 states: all-cause dementia, PD, and all-cause death. According to the boxes depicted in Fig. 2, the transitions were as follows: (*h1*) The number of individuals who are healthy and develop PD out of a total selected sample of 361,970 is 2423; (*h2*) the number of individuals who are healthy and develop dementia of any cause out of the same sample was 6018; (*h3*) the number of healthy individuals who died out of the total selected sample was 31,093; (*h4*) out of the 2423 individuals with PD, 314 also developed dementia of any cause, (*h5*) among the 2423 individuals with PD, 556 died; (*h6*) out of the 6018 individuals with all-cause dementia at follow-up, 2476 died. Figure S2 displays the transition survival probabilities for each of the four states. The survival rates for transitioning from states 2 and 3 (PD and dementia) are displayed in Figure S3. Both figures indicate that by the age of 80, a significant fraction of individuals would have experienced a transition from a healthy condition to one of the three remaining states, with approximately 1% transitioning to PD, 2% to dementia, and 18% to death. Additionally, around 90% of those with PD would have transitioned to either dementia or death states, while approximately 95% of individuals with dementia would have transitioned to death.

Table 3 presents the findings of both the fully adjusted model (model 2) and the reduced parametric Weibull and flexible Royston-Parmar survival models, which adjusted for a smaller set of covariates (model 1). These models assessed the relationships between POLYPH^+^ and the hazards of six distinct health transitions over time. POLYPH^+^, when defined as 2+ medications used, showed a positive relationship with the risk of transitioning from a state of health to PD, dementia, and death in both the reduced model but only from healthy to dementia and death for the fully adjusted models (i.e., transitions 2: HR=1.15, 95% CI: 1.07–1.23, *P*<0.001 and 3: HR=1.11, 95% CI: 1.08–1.09, *P*<0.001). Conversely, there was no detectable association found between POLYPH^+^ and transitions 4, 5, or 6. The association for transitions 2 and 3 (healthy to dementia and healthy to death) was significantly attenuated when comparing models 1 and 2, indicating that poor CVH (higher LE8_zrev_), co-morbidity, and self-rated health accounted for a large portion of these associations. The Weibull models and the Royston-Parmar model produced nearly identical results. Supplementary Table 2 presents a more comprehensive sensitivity analysis investigating all possible alternative distributions for parametric survival models. Using a higher threshold of 5+ vs. 0–4 medications used, similar though stronger associations were detected for the same transition, in addition to transition 1 in both reduced and fully adjusted models (healthy → PD) and transition 6 for the fully adjusted model (dementia → death).

**Table 3:** Parametric survival models (Weibull and flexible, Royston-Parmar) for the association between polypharmacy and six transitions between four states (healthy, PD, dementia, and death): UK Biobank 2006–2021

	Model 1^a^			Model 2^b^		
	HR	95% CI	*P*	HR	95% CI	*P*
Transition 1: Healthy → PD						
2+ vs. 0–1 medications						
Weibull	1.29	1.18–1.41	<0.001	1.08	0.98–1.19	0.13
Royston–Parmar	1.29	1.19–1.41	<0.001	*1.09*	*0.99–1.20*	*0.093*
5+ vs. 0–4 medications						
Weibull	1.45	1.33–1.58	<0.001	1.20	1.08–1.32	0.001
Royston–Parmar	1.45	1.32–1.58	<0.001	1.20	1.09–1.33	<0.001
Transition 2: Healthy → Dementia						
2+ vs. 0–1 medications						
Weibull	1.55	1.46–1.65	<0.001	1.15	1.07–1.23	<0.001
Royston–Parmar	1.56	1.46–1.65	<0.001	1.15	1.08–1.23	<0.001
5+ vs. 0–4 medications						
Weibull	1.81	1.71–1.91	<0.001	1.33	1.25–1.42	<0.001
Royston–Parmar	1.81	1.71–1.91	<0.001	1.34	1.26–1.42	<0.001
Transition 3: Healthy → Mortality						
2+ vs. 0–1 medications						
Weibull	1.56	1.52–1.60	<0.001	1.11	1.08–1.14	<0.001
Royston–Parmar	1.56	1.52–1.60	<0.001	1.12	1.09–1.15	<0.001
5+ vs. 0–4 medications						
Weibull	1.87	1.83–1.92	<0.001	1.30	1.26–1.34	<0.001
Royston–Parmar	1.87	1.83–1.92	<0.001	1.31	1.27–1.35	<0.001
Transition 4: PD → Dementia						
2+ vs. 0–1 medications						
Weibull	1.08	0.84–1.40	0.54	1.03	0.78–1.37	0.82
Royston–Parmar	1.09	0.84–1.41	0.52	1.04	0.78–1.37	0.80
5+ vs. 0–4 medications						
Weibull	1.01	0.80–1.29	0.92	0.94	0.71–1.23	0.64
Royston–Parmar	1.01	0.80–1.29	0.92	0.93	0.71–1.23	0.62
Transition 5: PD → Mortality						
2+ vs. 0–1 medications						
Weibull	1.00	0.83–1.21	0.98	0.99	0.81–1.21	0.91
Royston–Parmar	1.00	0.83–1.20	0.98	0.98	0.80–1.21	0.86
5+ vs. 0–4 medications						
Weibull	1.10	0.92–1.32	0.27	1.11	0.90–1.36	0.33
Royston–Parmar	1.11	0.93–1.33	0.24	1.11	0.91–1.37	0.30
Transition 6: Dementia → Mortality						
2+ vs. 0–1 medications						
Weibull	1.05	0.96–1.15	0.29	1.09	0.98–1.20	0.11
Royston–Parmar	1.04	0.95–1.14	0.29	1.09	0.98–1.20	0.12
5+ vs. 0–4 medications						
Weibull	*1.08*	*1.00–1.17*	*0.057*	1.14	1.04–1.26	0.006
Royston–Parmar	*1.08*	*1.00–1.17*	*0.056*	1.14	1.04–1.26	0.006

*Abbreviations*: *CI*, confidence interval; *HR*, hazard ratio; *LE8*, Life’s Essential 8; *PD*, Parkinson’s disease; *SES*, socio-economic status z-score; *SES*_*res*_, residual socio-economic status (z-score) from linear model of SES → TDI; *TDI*, Townsend Deprivation Index; *UK*, United Kingdom

^a^Model 1 was adjusted for baseline age, sex, race/ethnicity (non-White vs. White), household size, TDI z-score, and SES residual z-score

^b^Model 2 was adjusted for baseline age, sex, race/ethnicity (non-White vs. White), household size, LE8_zrev_, TDI z-score and SES residual z-score, co-morbidity index, and self-rated health

**Fig. 3:**
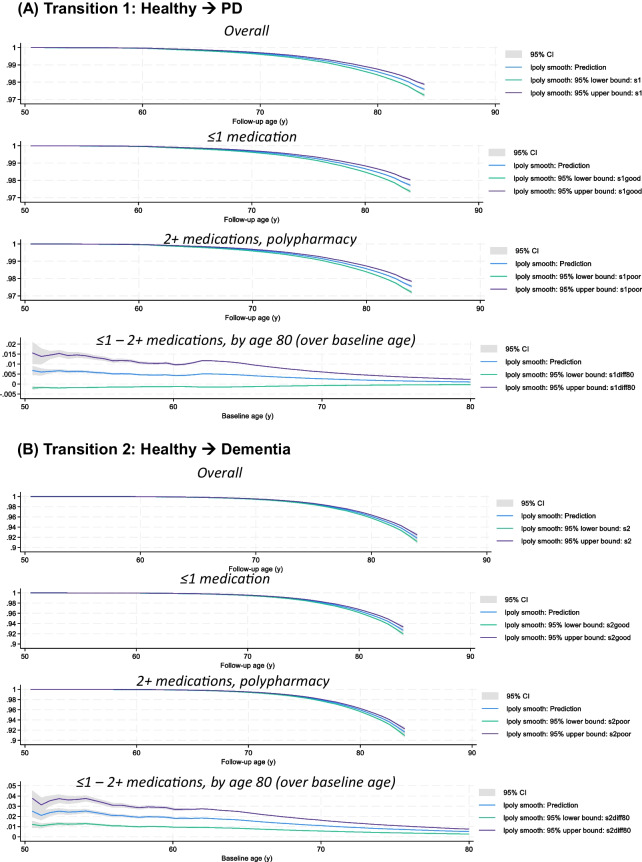

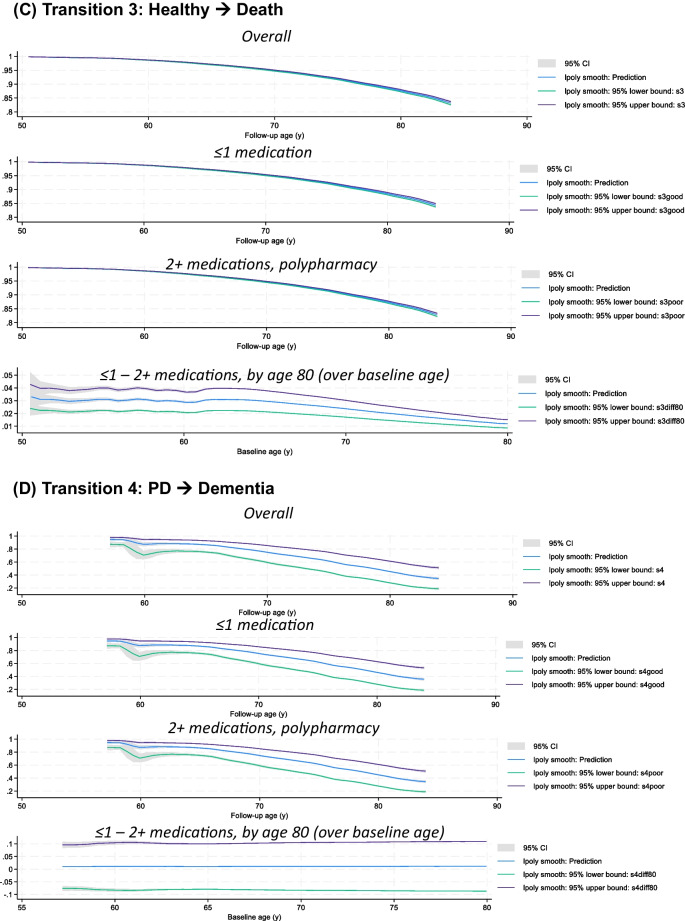

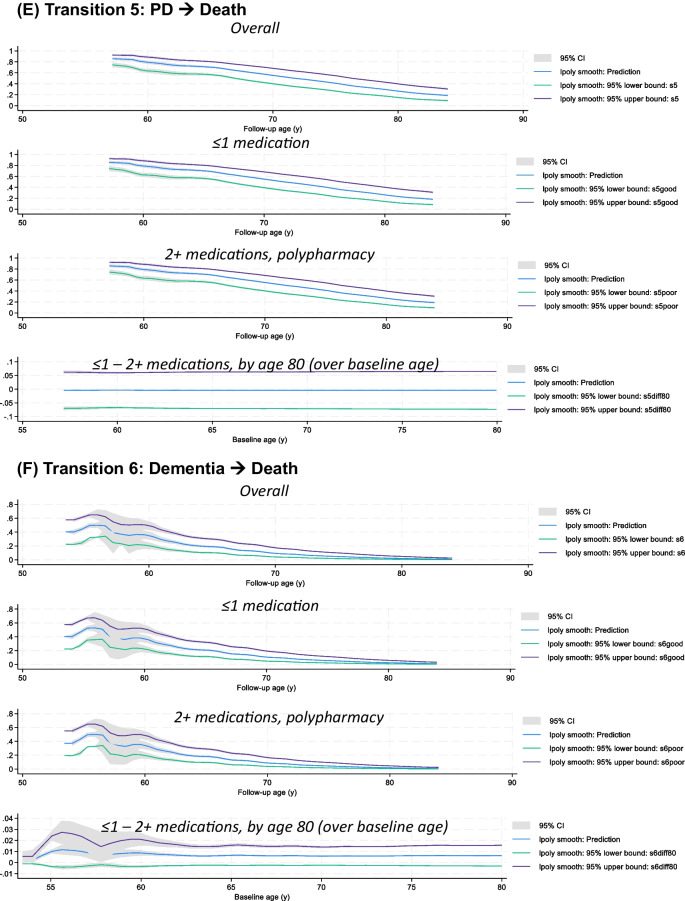
Transition survival probability for all 4 states, overall, among POLYPH^−^, POLYPH^+^, and differences in survival (POLYPH^−^ - POLYPH^+^): UK Biobank 2006–2021. *Abbreviations*: PD, Parkinson’s disease; lpoly, local polynomials. *Notes:* Predicted survival probabilities and difference in survival probabilities with their 95% CI obtained from a Weibull model using *stmerlin* command in Stata. Local polynomials are used for smoothing. See “Methods” section for details

The fully adjusted Weibull model (model 2 in Table 3) was utilized to predict survival probabilities for each of the six transitions, as shown in Fig. 3, focusing on the main exposure for POLYPH (2+ medications vs. 0–1 medications). Following the setting of POLYPH to 0 (i.e., POLYPH^−^) and 1 (POLYPH^+^), these survival probabilities were likewise predicted. The survival probability differences between these two exposure levels were also post-estimated, while maintaining the other covariates as they were observed in the study population. This difference was represented across baseline ages, which might vary from 50 to 79 years old, at the follow-up age of 80 years old. Based on estimations with local polynomials, survival in for transitions 2 and 3 between POLYPH^−^ and POLYPH^+^ differed significantly by the age of 80 years, with POLYPH^+^ significantly lowering survival for these two transitions (healthy → dementia and healthy → death). Supplementary Table 3 also provides descriptive information on these variations in survival. The output is available at https://github.com/baydounm/UKB-paper16-supplementarydata.

**Fig. 4:**
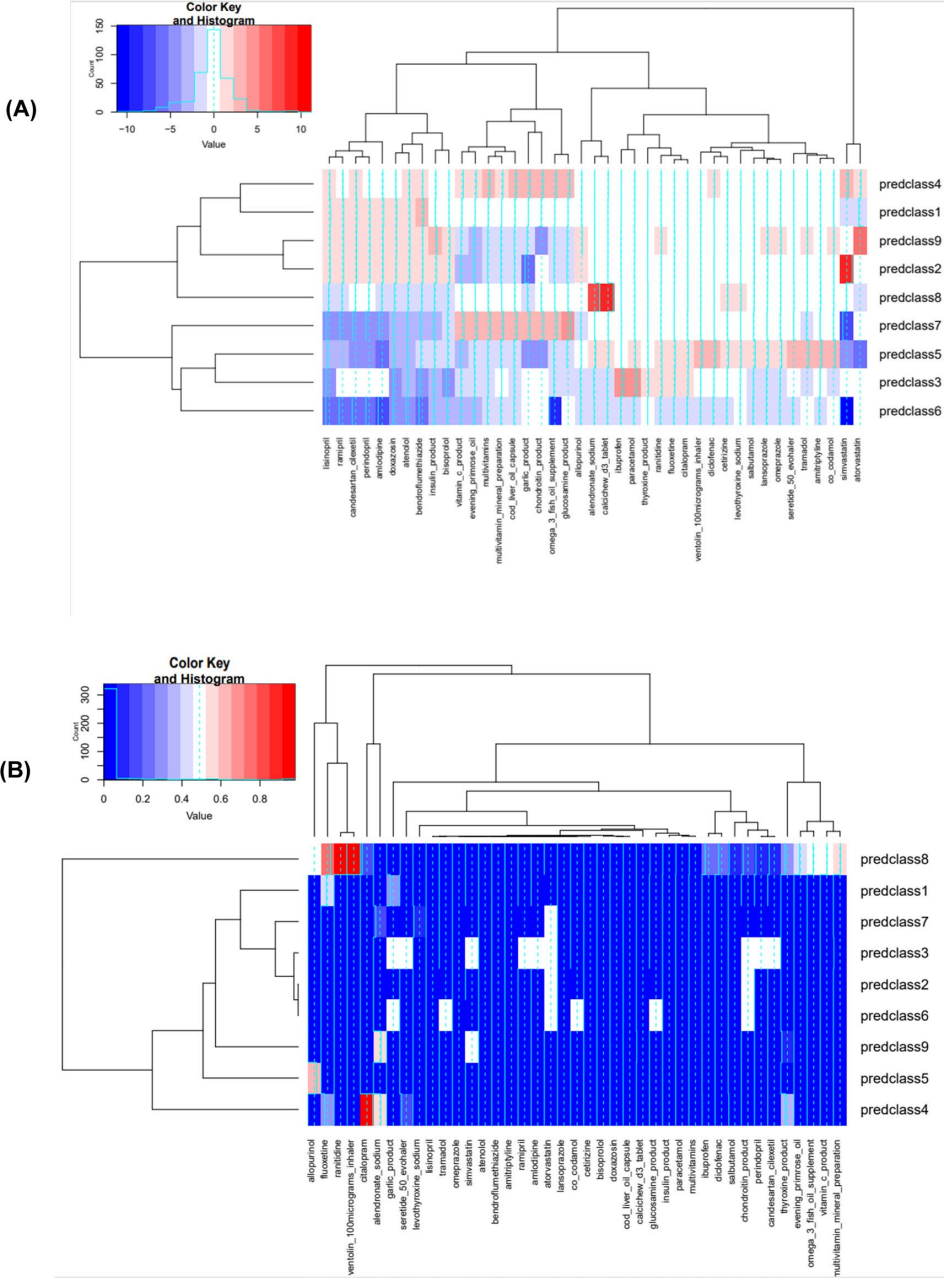
Lnodds of latent class membership for most commonly used medications: UK Biobank 2006–2021. (**A**) Lnodds; (**B**) p-value of Lnodds. *Abbreviations*: predclass, latent class membership (0 = no, 1 = yes)

Examining each commonly used medication and generating predicted group membership using LCA, 9 groups were generated that could be labeled using a bivariate logistic regression with each medication. The findings from these series of logistic regression models are shown in Fig. 4, by comparing Ln(odds ratios) across those LCs for each of the 48 commonly used specific medications, using a heat map. Following this, a series of GSEM models were carried out aiming at examining the direct association between each of these LCs on the transitions of interest (healthy → dementia; healthy → mortality) that are not explained by POLYPH, adjusting for various exogenous variables. POLYPH was defined using the two cutoffs of interest (2+ and 5+) in Table 4 and S4, respectively. For both cutoffs, LC3 was inversely associated with the healthy → dementia transition, while LC9 had a positive association with this same transition. For the healthy → mortality transition, LCs that stood out for both cutoffs consistently were LC3 (−), LC4 (−), LC6 (+), LC7 (−), LC8 (+), and LC9 (+). With few exceptions (e.g., LC3, LC6) whereby membership in a latent class was inversely related to POLYPH, latent classes were generally positively associated with both POLYPH measures (2+ and 5+ definitions). Combining information from Fig. 4 and Tables 4 and S4, several medications and supplements stood out as potentially having an inverse association with both transitions that were not explained by POLYPH, namely increased use of various supplements including omega-3 fatty acids, multivitamins, and glucosamine coupled with reduced use of simvastatin (LC7). Other medications or combinations of medications that may potentially have a deleterious effect on both mortality and dementia risk were reflected in LC9, without being explained by by POLYPH. LC9 reflects increased use of atorvastatin and insulin product and reduced use of chondroitin product.

**Table 4:** Latent classes of commonly used medications vs. transitions 2 and 3, as mediated through polypharmacy as defined by a cutoff of 2+ medications (POLYPH^2+^): direct and indirect effects: UK Biobank 2006–2021

LC	Outcome	LC → POLYPH^2+^			LC → Outcome			POLYPH^2+^ → Outcome		
		β	(SE)	*P*	β	(SE)	*P*	β	(SE)	*P*
1	Healthy to dementia	1.31	0.02	<0.001	−0.04	0.04	0.29	0.14	0.03	<0.001
2	Healthy to dementia	1.90	0.02	<0.001	−0.02	0.03	0.58	0.14	0.03	<0.001
3	Healthy to dementia	22.0	__	__	−0.06	0.08	0.50	0.14	0.03	<0.001
4	Healthy to dementia	5.48	0.29	<0.001	−0.04	0.08	0.61	0.14	0.03	<0.001
5	Healthy to dementia	3.08	0.05	<0.001	−0.04	0.06	0.55	0.14	0.03	<0.001
6	Healthy to dementia	−3.58	0.01	<0.001	−0.05	0.04	0.21	0.10	0.04	0.02
7	Healthy to dementia	3.30	0.02	<0.001	−0.13	0.05	0.01	0.16	0.03	<0.001
8	Healthy to dementia	22.73	__	__	0.09	0.21	0.68	0.14	0.03	<0.001
9	Healthy to dementia	2.40	0.04	<0.001	0.27	0.04	<0.001	0.11	0.03	<0.001
1	Healthy to mortality	1.31	0.02	<0.001	0.01	0.02	0.462	0.10	0.02	<0.001
2	Healthy to mortality	1.90	0.02	<0.001	−0.02	0.02	0.241	0.11	0.02	<0.001
3	Healthy to mortality	22.0	__	__	−0.18	0.04	<0.001	0.12	0.02	<0.001
4	Healthy to mortality	5.48	0.29	<0.001	−0.12	0.04	0.001	0.11	0.01	<0.001
5	Healthy to mortality	3.08	0.05	<0.001	0.04	0.03	0.167	0.10	0.02	<0.001
6	Healthy to mortality	−3.58	0.01	<0.001	0.08	0.02	<0.001	0.16	0.02	<0.001
7	Healthy to mortality	3.30	0.02	<0.001	−0.21	0.03	<0.001	0.13	0.02	<0.001
8	Healthy to mortality	20.50	__	__	0.42	0.09	<0.001	0.10	0.01	<0.001
9	Healthy to mortality	2.40	0.04	<0.001	0.11	0.02	<0.001	0.10	0.02	<0.001

Abbreviations: LC, latent class; P, P-value; POLYPH, polypharmacy; SE, standard error; UK, United Kingdom

## Discussion

We examined the relationship between POLYPH and the risk of all-cause dementia, PD, and all-cause mortality in the UK Biobank. In the fully adjusted Cox proportional hazards model, POLYPH^+^ was associated with an increased risk of all-cause dementia and mortality in both sexes, with a stronger association observed in males for dementia. The fully adjusted Weibull multistate model showed that POLYPH+ significantly reduced survival for transitions from healthy to dementia and healthy to death. A higher threshold of 5 or more medications further increased the risk for transitions from healthy to PD and from dementia to death. Using latent class analysis and generalized SEM models, certain combinations of medications, including omega-3 fatty acids and multivitamins, were inversely related to these transitions, independent of polypharmacy, while other combinations were positively associated with these outcomes.

Previous studies have associated polypharmacy with dementia (5, 6, 14) as well as prognosis among individuals diagnosed with dementia (7). A study investigating the relationship of polypharmacy with dementia risk in older adults in Taiwan showed that the incidence of dementia increased with the number of medications used and age and that comorbidities (e.g., cerebrovascular disease, diabetes mellitus, chronic kidney disease, hypertension) were also significant predictors of dementia risk (6). Using the South Korean National Health Insurance Service sample cohort database, a study examining the relationship of polypharmacy with dementia risk revealed that polypharmacy was correlated with comorbidities and potentially inappropriate medications, with significant interactions of polypharmacy with specific medications and comorbid conditions (14). There were increasing odds of dementia with increasing level of polypharmacy levels, particularly in the absence of medications and comorbid conditions that interact with polypharmacy (14). A study in South London found that patients with dementia who were prescribed 4–6 or ≥7 medications had a higher risk of emergency department attendance, hospitalization, unplanned hospital admission, and death within 2 years (7). The study also found a dose-response relationship with each additional drug, increasing the risk of emergency department attendance and mortality by 5% and hospitalization by 3% (7). Finally, a systematic review and meta-analysis whereby seven studies were included summarized existing literature on the association between polypharmacy and dementia, revealing a strong association between polypharmacy and dementia, while also suggesting that excessive polypharmacy was strongly associated with dementia (5). Here, we also found that polypharmacy (5+ medications) was associated with a greater risk of transitioning from healthy to PD. Polypharmacy is particularly relevant for PD patients, as multiple medications are often given to treat both non-motor and motor symptoms as well as PD patients often have additional comorbidities that require medications (16). Many medications are beneficial for PD (16). For example, non-aspirin nonsteroidal anti-inflammatory drugs (NSAIDs) use has been linked to a reduced risk for PD (15). However, with an increased number of medications, there is a higher risk of drug interactions in PD patients (16), which requires precise drug management in these patients. Nevertheless, a recent cross-sectional study of newly diagnosed hospitalized PD patients reported that polypharmacy was associated with cognitive decline (19). However, there is little data on the impact of polypharmacy on transitioning from healthy to PD. Our findings indicate limited association between polypharmacy and the transition from healthy → PD, particularly after adjustment for potential confounders, possibly due to the high consumption of NSAIDs in this population.

A recent meta-analysis encompassing 24 cohort studies reported a positive association between polypharmacy (defined as the use of five or more medications) and mortality in older adults, with a relative risk of 1.28 (95% CI 1.19–1.39) (13). Complementary findings come from a Korean study, not included in the meta-analysis above, which reported a similar 25% increased risk of death (34). Additionally, a German study involving a population-based cohort of older adults found that polypharmacy was associated with a two-fold increase in non-cancer death risk (35). In the USA, among elderly patients with chronic kidney disease, using five or more medications was linked to a 27% higher risk of all-cause mortality and a 39% higher risk of cardiovascular death (36).

When examining commonly used medications and supplements, several stood out as potentially protective against dementia and mortality risk, independently of polypharmacy. Previous studies show that in fact many of these medications and supplements may have a protective effect as shown in Online Supplementary Material 3 (full literature review with references). Those include multivitamins, omega-3 fatty acids, glucosamine, and chondroitin sulfate. In summary, while multivitamins have inconclusive evidence of beneficial or harmful effects on cognitive outcomes, they are commonly used for indications other than PD. Omega-3 polyunsaturated fatty acids were shown to have positive effects on cognitive function and to reduce the progress of PD. Glucosamine supplement intake has been shown to lower the risk of dementia and lower mortality for all causes, cancer, cardiovascular disease, respiratory, and digestive diseases. Chondroitin sulfate proteoglycan-related enzymes have been linked to cognitive function, with regular intake associated with reduced all-cause and cardiovascular disease mortality. Thus, these medications may be good targets for further large randomized controlled trials.

Our study has several strengths. Utilizing data from the UK Biobank allowed us to analyze a large sample of over 360,000 individuals with up to 15 years of follow-up while accounting for a broad range of confounders. In addition to traditional Cox proportional hazards models, we employed multistate models to capture the complex progression between health states as well as LCA of different medications, providing a more nuanced assessment of the pathways through which polypharmacy and patterns of medication use may impact health outcomes. However, several limitations are worth noting. First, the parent study offers a large and comprehensive dataset pertaining to the health of a sample of UK participants who resided near 22 study assessment centers. However, it cannot be thought of as being fully representative of the general middle-aged and older adult UK population. In fact, selected participants tend to be healthier, more affluent, and less ethnically diverse than the general population, which may further limit the generalizability of our findings to broader, more diverse populations. Additionally, as with all observational studies, measurement error in outcomes and exposures, particularly in self-reported data, could introduce bias. Finally, multistate models offer advantages in capturing disease progression compared to Cox and parametric models with a single disease outcome. However, the former models rely on several assumptions that include the Markov assumption, which may not fully reflect the true nature of health transitions.

Polypharmacy is associated with lower survival rates among individuals with and without dementia. The elevated use of medications appears to be linked to a faster rate of all-cause mortality and an earlier onset of dementia, even after adjusting for comorbidities. These findings highlight the importance of careful prescribing practices and regular medication reviews to mitigate potential harm, particularly in vulnerable populations such as older adults with multiple chronic conditions. Further studies in similar populations are needed to replicate and extend these findings.

## Supplementary information

Below is the link to the electronic supplementary material.

Supplementary file1 (PDF 662 KB)

## Data Availability

The data used here can be accessed through the UK Biobank resource (www.ukbiobank.ac.uk). Our present study is part of an approved project application 77963.
