# Supplementary material for: Polypharmacy and its association with dementia, Parkinson’s disease, and mortality risk in UK adults: a multistate modeling approach
Source: GeroScience. 2025 Mar 13;47(3):4349–67. doi: 10.1007/s11357-025-01586-w (PMC12181516; doi:10.1007/s11357-025-01586-w)
Supplement: Supplementary file 1 — Supplementary file1 (PDF 662 KB) [file 11357_2025_1586_MOESM1_ESM.pdf]

Polypharmacy and its association with dementia, Parkinson's Disease and mortality risk in UK adults: a multistate modeling approach

Jordan Weiss et al.

ONLINE SUPPLEMENTARY METHODS 1

Detailed steps of the multistate modeling process are provided below along with description of sensitivity analyses: (i) We utilized Stata's *msset* command to arrange our dataset, incorporating 3 events of interest along with their corresponding age at occurrence. Additionally, we specified the age at baseline assessment, which corresponds to the initial instance of the UKB dataset. Events included incidence of PD or dementia, and death from any cause (considered as the last event). Individuals who did not experience any events were classified as "healthy." A transition transformation matrix was produced using the *msboxes* command, which included a multistate set of boxes with four states: "Healthy", "PD", "Dementia", and "Deaths." (ii) The data was subsequently organized in long format for all 3 events combined, using _trans1 through $\_trans_k$ to isolate each transition from the identified group of k transitions. (iii) The Aalen-Johansen estimates of transition probabilities were calculated, together with their 95% confidence intervals. These estimates represent the likelihood of being in each of the four states as age progresses, as well as the likelihood of transitioning from PD and Dementia to other states. The *msaj* command was employed for this purpose. The data points were graphically represented using line plots with step functions (1).

(iv) Parametric survival models were estimated using POLYPH as the primary variable of interest. The potentially confounding factors included age at baseline, sex, race (Non-White vs. White), household size, TDI z-score, SES_{res}, LE8zrev, the co-morbidity index and self-rated health. The models were primarily based on the Weibull distribution and were constructed using the *stmerlin* command (2). The efficacy of this parametric model was subsequently assessed by comparing it to alternative models employing different distributions (see to **supplemental Table 1** for further information). The model with the lowest AIC and/or BIC was compared to the Weibull model in order to assess the relationship between the TDI z-score and the outcome of interest. The models are constructed for every identified *k* transition. Among other alternatives, the Royston-Parmar flexible parametric model was also taken into consideration (3). (v) Survival probabilities were estimated for each transition and associated model. The estimation was done

twice: first without considering covariates, and second by assigning values of POLYPH as either 0 (indicating 0-1 medications used) or 1 (indicating 2+ medications used). The estimation was performed using the *stmerlin* postestimation predict command and options, with a Weibull distribution (2). The model-based estimated survival probabilities for each transition were shown, together with their 95% confidence intervals, using Kernel-weighted local polynomial smoothing throughout the age of follow-up (4). By extending the age at follow-up to 80 years, we were able to estimate the difference in survival probability between individuals who screen negative vs. positive for POLYPH for each transition. The prediction was accompanied by a 95% confidence interval (2). We applied a comparable smoothing technique using local polynomials on baseline age values that were limited to a range of 50 years to 79 years (4). Greenwood pointwise standard errors were computed to determine the predicted survival probabilities and differences in survival probability (2). The study provides descriptive statistics, including the median and interquartile range (IQR), for point estimates. These statistics are reported mainly for the predicted differences at age 80y for each transition.

(vi) Furthermore, a sensitivity analysis was performed to compare the fully adjusted parametric model with different distributional specifications.

(vii) In order to examine further which groups of medications were explaining any association between exposure and outcome, UKB field 20003 was used for instance 0 (or baseline assessment) covering an array of 48 possible specific medications. The most commonly used medications were defined by an overall frequency of 5000 or more in the entire UK Biobank study (i.e. out of the 500K participants). These data were used to conduct a latent class analysis (LCA) and generate probabilities of membership in each of these latent classes. These probabilities of group membership were then labelled based on their relationship to the selected medications used and heatmaps were used for visualization. Importantly, they were entered into a mediation analysis whereby POLYPH was the main exposure, each latent class group membership probability (converted to Ln(odds)) was one of several mediators, and the outcome was the time-to-event to each of uncovered health state transitions. This analysis was conducted using generalized structural

equations (GSEM) models, in an attempt to explain detected associations between $POLYPH^+$ and any of the outcomes of interest at a type I error of 0.05. Exogenous variables in the GSEM model included all potentially confounding covariates listed below in the "Covariates" section. More details regarding both the LCA and GSEM are provided in **supplementary Method 2**.

(viii) A secondary definition for POLYPH with a threshold of 5^+ medications instead of 2^+, was used in part of the analysis, namely for part iv and vii.

Supplementary Table 1.

Model	Description
Weibull Distribution	Models failure times characterized by a monotonic hazard function. The shape parameter (β) allows flexibility.
Exponential Distribution	Models constant hazard over time, with a memoryless property such that future survival depends only on the current time.
Gompertz Distribution	Models a hazard that increases exponentially over time. Useful for biological processes with aging effects.
Piecewise Exponential with 3 Knots	Models the hazard rate as constant within predefined intervals (knots), allowing for different hazard rates in each interval.
Restricted Piecewise	Similar to the piecewise exponential model but with the constraint that hazard rates do not increase across intervals.
Restricted Cubic Spline	Models complex hazard functions with smooth transitions between segments defined by knots, offering greater flexibility.

Supplementary Table 2. Parametric survival models for the association between $POLYPH^{2+}$ and six transitions between four states (healthy, PD, dementia, and death): UK Biobank 2006-2021[a]

	Transition 1 Healthy→PD	Transition 2 Healthy→Dementia	Transition 3 Healthy→Mortality	Transition 4 PD→Dementia	Transition 5 PD→Mortality	Transition 6 Dementia→Mortality
Model	Coef (SE) [P]	Coef (SE) [P]	Coef (SE) [P]	Coef (SE) [P]	Coef (SE) [P]	Coef (SE) [P]
Weibull Distribution	0.075 (0.049) [0.13] BIC=39206	0.138 (0.034) [**<0.001**] BIC=77892	0.105 (0.015) [**<0.001**] BIC= 325286	0.032(0.143) [0.82] BIC=2615	-0.013 (0.105) [0.91] BIC=3971	0.083(0.053) [0.11] BIC= 13309
Exponential Distribution	0.108 (0.05) **[0.028]** BIC=48853	0.179 (0.034) [**<0.001**] BIC=102895	0.132 (0.015) [**<0.001**] BIC= 426575	0.047(0.143) [0.75] BIC= 2621	0.005 (0.105) [0.96] BIC=3987	0.094 (0.053) [0.075] BIC=13329
Gompertz Distribution	0.073 (0.049) [0.14] BIC=39274	0.136 (0.034) [**<0.001**] BIC=77931	0.103 (0.015) [<0.001] BIC= 32581	0.032(0.143) [0.83] BIC=2616	-0.014(0.105) [0.89] BIC= 3969	0.083 (0.053) [0.12] BIC=13308
Piecewise Exponential with 3 Knots	0.108 (0.049) **[0.028]** BIC=49670	0.179 (0.034) [**<0.001**] BIC=102452	0.132 (0.015) [**<0.001**] BIC= 424280	0.047(0.144) [0.75] BIC= 2621	0.005 (0.105) [0.96] BIC=3987	0.094 (0.053) [0.075] BIC=13329
Restricted Piecewise	0.083 (0.049) [0.093] BIC=39052	0.144 (0.033) [**<0.001**] BIC=77636	0.111 (0.015) [**<0.001**] BIC= 322563	0.036(0.143) [0.80] BIC= 2637	-0.019 (0.106) [0.86] BIC= 3987	0.082 (0.053) [0.12] BIC=13333
Restricted Cubic Spline	0.082 (0.049) [0.096] BIC=39057	0.144 (0.034) [**<0.001**] BIC=77648	0.110 (0.015) [**<0.001**] BIC=322741	0.034(0.143) [0.81] BIC= 2637	-0.017 (0.105) [0.87] BIC=3988	0.083 (0.052) [0.12] BIC= 13334

Abbreviations: BIC=Bayesian Information Criterion; Coef = Coefficient; $LE8_{zrev}$=z-scored Life's Essential 8 total score, multiplied by -1; P = p-value; PD = Parkinson's Disease; POLYPH=Polypharmacy; SE = standard error; SESres=residual from linear regression of SES z-score on TDI z-score; TDI=Townsend Deprivation Index; UK=United Kingdom.

[a] All parametric survival models adjusted for age, sex, race, SES_{res} , TDI, household size, and $LE8_{zrev}$. Values are estimated $Log_e(HR)$ with SE and associated p-value for null hypothesis that $Log_e(HR)=0$. Alternative distributions were tested and compared using BIC.

Supplementary Table 3. Descriptives of predicted differences (medians of prediction, lower 95% CI limit and upper 95% CI limit) in survival probability for each of 6 transitions, comparing POLYPH$^-$ vs. POLYPH$^+$ with age at event set at 80y[a]

	Transition 1	Transition 2	Transition 3	Transition 4	Transition 5	Transition 6
	"Healthy"→ PD	"Healthy" → Dementia	"Healthy"→ Death	PD→Dementia	PD→Death	Dementia→Death
Prediction	+0.0019	0.0080	0.019	+0.011	-0.004	+0.005
LCL	-0.0005	0.0041	0.014	-0.088	-0.075	-0.003
UCL	+0.0043	0.012	0.024	+0.111	+0.066	+0.014

[a] *Based on fully adjusted Weibull model with POLYPH as main exposure, and adjusted for age, sex, race, household size, TDI z-score, SESres, and LE8zrev.*

Abbreviations: PD = Parkinson's Disease; POLYPH=Polypharmacy; SD=Standard Deviation; SE = standard error; TDI=Townsend Deprivation Index; UK=United Kingdom.

ONLINE SUPPLEMENTARY METHODS 2. Latent class analysis and generalized SEM

Latent class analysis in R

Latent Class Analysis (LCA) is a statistical method employed to discern concealed classes within a population through observed categorical data(5, 6, 7). The *poLCA* package in R offers an extensive array of tools for estimating latent class models, enabling users to specify the required number of classes, apply the model to the dataset, and evaluate its fit using metrics such as the Akaike Information Criterion (AIC) and Bayesian Information Criterion (BIC)(5, 6, 7). The *poLCA* function streamlines model specification by enabling users to delineate the formula for observable variables and the quantity of classes(5, 6, 7). The system employs Expectation-Maximization (EM) techniques to determine the probability of class membership and item answers(5, 6, 7). The application additionally incorporates visualization tools for analysis(5, 6, 7). Below are the key equations on the basis of which latent class analysis for medication use class membership is determined, assuming a total of *J* medications that were considered commonly used based on a cutoff for frequency of 5,000 in the largest possible UKB sample.

(Eq. 1.1) *Latent class probabilities*:

$$\boldsymbol{P(Z_i = k) = \pi_k}$$

(Eq. 1.2) *Conditional probabilities*:

$$P(X_{ij=x}|\, Z_i = k) = \theta_{jk}(x)$$

(Eq. 1.3) *Joint probability*:

$$\boldsymbol{P(X_i|Z_i = k\,) = \prod_{j=1}^{J} P(X_{ij}\,|Z_i = k)}$$

(Eq. 1.4) *Marginal likelihood*:

$$\boldsymbol{P(X_i) = \sum_{k-1}^{K} \pi_K \prod_{j=1}^{J} P(X_{ij} \mid Z_I = k\ )}$$

(Eq. 1.5) *Log-likelihood*:

$$\boldsymbol{L(\theta) = \sum_{i-1}^{N} log(\sum_{k-1}^{K} \pi_k \prod_{j=1}^{J} P(X_{ij}| Z_i \ = \ k)}$$

(Eq. 1.6) *Posterior probability of class membership*:

$$P(Z_i \ = k \mid X_i) = \frac{\pi_k \prod_{j=1}^{J} P(X_{ij} \mid Z_i = k)}{\sum_{k=1}^{K} \pi_k \prod_{j=1}^{J} P(X_{ij} \mid Z_i = k)}$$

Generalized SEM in Stata

This appendix offers an elaborate manual on performing Generalized Structural Equation Modeling (SEM) in Stata, with the condition that the ultimate outcome Y adheres to a Weibull distribution. The model comprises a primary binary exposure variable X, a continuous mediator M, and many exogenous variables Zi, which are permitted to predict the ultimate outcome, the mediator, and the primary exposure variable.

1. Model Specification

The generalized SEM can be conceptualized with the following structure:

1. **Final Outcome Model:**

$$Y \sim \text{Weibull}(\lambda, k)$$

$$\log(\lambda) = \beta_0 + \beta_1 X + \beta_2 M + \sum_{i=1}^{p} \beta_{2+i} Z_i$$

2. **Mediator Model:**

$$\Pr(M = 1) = \frac{\exp(\gamma_0 + \gamma_1 X + \sum_{i=1}^{p} \gamma_{1+i} Z_i)}{1 + \exp(\gamma_0 + \gamma_1 X + \sum_{i=1}^{p} \gamma_{1+i} Z_i)}$$

3. **Exposure Model:**

$$\Pr(X = 1) = \frac{\exp(\delta_0 + \sum_{i=1}^{p} \delta_i Z_i)}{1 + \exp(\delta_0 + \sum_{i=1}^{p} \delta_i Z_i)}$$

Here, λ is the scale parameter controls the expected survival time, and log(λ) is the log of time to event, k is the shape parameter of the Weibull distribution, β, γ, and δ are the coefficients, and ϵ_M is the error term for the mediator.

In hazard function formulation, the Weibull model is as follows:

$$h(t) = \frac{p}{\lambda} \left(\frac{t}{\lambda} \right)^{p-1}$$

where: t is the time, p is the shape parameter, λ is the scale parameter.

The shape parameter p in the Weibull distribution controls the distribution's form (exponential when p=1; monotonic increasing or decreasing hazard for other values).

2. Data Preparation

Ensure that the dataset is correctly formatted with the variables Y (continuous), X (binary), M (continuous), and Zi (exogenous variables).

3. Stata Code for Generalized SEM

Below is the Stata code to fit the specified generalized SEM:

```
* Load the data
use your_dataset.dta, clear


* Define the model
gsem (X <- Z1 Z2 Z3, logit) ///
     (M <- X Z1 Z2 Z3, logit) ///
     (Y <- X M Z1 Z2 Z3, weibull)
```

Example:

```
**Class 1**
gsem (_t <- POLYPH NonWhite AGE SEX householdsize ztownsend SESres zLE8_TOTALSCOREinv
comorbid srhbr predclass1, family(weibull, failure(_d) ) link(log) nocapslatent) ///
(NonWhite -> POLYPH, family(binomial) link(logit)) ///
(AGE -> POLYPH, family(binomial) link(logit)) ///
(SEX -> POLYPH, family(binomial) link(logit)) ///
(householdsize -> POLYPH, family(binomial) link(logit)) ///
(ztownsend -> POLYPH, family(binomial) link(logit)) ///
```

```
(SESres -> POLYPH, family(binomial) link(logit)) ///
(zLE8_TOTALSCOREinv -> POLYPH, family(binomial) link(logit)) ///
(comorbid -> POLYPH, family(binomial) link(logit)) ///
(srhbr -> POLYPH, family(binomial) link(logit)) ///
(predclass1  -> POLYPH  , family(binomial) link(logit)) ///
if sample_final==1 & _trans2==1, nocapslatent method(ml)



* Output the results
estat ic, all
estat gof, stats(all)
```

4. Model Explanation

1. **Binary Exposure Model:** The exposure X is modeled through a logistic regression where X is predicted by the exogenous variables Zi .
2. **Mediator Model:** The binary mediator M is regressed on the exposure X and the exogenous variables Zi.
3. **Final Outcome Model:** The final outcome Y, assumed to follow a Weibull distribution, is modeled as a function of the exposure X, the mediator M, and the exogenous variables Zi.

5. Model Fit and Diagnostics

- The estat ic command is used to obtain information criteria (AIC and BIC) for model comparison.
- The estat gof command is used to obtain goodness-of-fit statistics for assessing the model fit.

6. Interpretation of Results

- The coefficients β1 and β2 indicate the direct effects of X and M on Y, respectively.
- The coefficients γ1 and δi provide insights into how the mediator and exposure are influenced by the exogenous variables.
- Assess the significance and magnitude of the coefficients to understand the relationships within the model.

Researchers can utilize generalized SEM (*gsem* command) in Stata to study intricate associations that involve a Weibull-distributed outcome, a binary exposure, a binary mediator, and many exogenous variables by adhering to these steps. In addition, indirect effects through M can be estimated and statistical significance assessed at a type I error rate of 0.05 assessed using non-linear combinations, namely the products of β_1 and γ_1, using *nlcom*. Standard error associated with this non-linear combination is estimated using the delta method. Nevertheless, in our present study, the direct effect of X on Y is of primary interest.

Main source: Stata and R manuals and help

Other sources: (5, 6, 8, 9)

ONLINE SUPPLEMENTARY MATERIALS 3: LITERATURE REVIEW ON SELECT COMMONLY USED MEDICATIONS IN RELATION TO NEURODEGENERATIVE DISEASE AND MORTALITY

candesartan_cilexetil

Candesartan cilexetil is a drug often used for arterial hypertension, however, it has been shown to produce positive effects for dementia, and other chronic conditions (10). In rat models, Candesartan cilexetil also showed a positive effect in models of Parkinson's disease, protecting dopaminergic neurons involved in blocking endoplasmic reticulum stress by inhibiting ATF4-CHOP-Puma pathway activation (11). Candesartan cilexetil effect in treating chronic conditions has been shown as protective against cardiovascular death, reduced hospitalizations, and all-cause mortality, however, blood pressure, serum potassium, and creatinine levels should be monitored (12, 13).

doxazosin

Doxazosin is a medication used for the treatment of urinary tract symptoms (14); however, it has been considered a potentially inappropriate medication for older adults and is often prescribed (15). Although doxazosin is often prescribed, some evidence shows an effect on reducing dementia with Lewy bodies in men (16). Additionally, some evidence showed a potential risk reduction in developing Parkinson's disease and slowing motor symptoms (17, 18). One study investigating in-hospital mortality from COVID-19 revealed doxazosin relative risk reduction for death of 74% (odds ratio 0.23; 95% CI 0.03-0.94; $p = 0.028$) (19).

Insulin

Studies of insulin and cognitive function have pointed to resistance to insulin (20, 21, 22). Insulin resistance and cognition studies have suggested that resistance may be a feature of Alzheimer's disease and cognitive impairments (20). It has recently been noted that dysfunction of insulin signaling can influence brain function, therefore increasing the risk of Alzheimer 's-related biomarkers like an increased number of beta-amyloid plaques and tau tangles (21). Also, insulin signaling is involved with miR-193b-3p/PGC-1α pathway, which plays an anti-inflammatory role during the early stages of Parkinson's disease (23). Triglyceride-glucose index, a validated marker for insulin resistance, was shown that at higher levels, it was associated with an increased risk of all-cause mortality (24), and an increased risk of heart failure for both men and women (25).

lansoprazole

Lansoprazole has been shown in animal models to improve locomotor activity, reduce tau, and increase the extent of phosphorylated tau-positive areas (26). In human imaging studies, (^{18}F) (18)N-methyl lansoprazole brain retention was low in mild-cognitive impaired/Alzheimer's disease patients despite a high affinity for tau in vitro (27). Lansoprazole, at new dosage levels, may be appropriate for patients with dysphagia, especially for the elderly who are neurologically impaired, such as Parkinson's disease, and reduced mortality (28). Additionally, lansoprazole appeared as a binding site for all fibrils for surface plasmon resonance and immunofluorescence staining in the brain for pS129-αSyn positivity in Parkinson's disease patients (29).

lisinopril

lisinopril has an 18-year cumulative incidence rate of 31.4% in Alzheimer's disease; however, this rate was nearly identical to chlorthalidone (30.5%) and amlodipine (31.1%) (30). Early onset androgenetic alopecia among men showed lower efficacy with lisinopril and other drugs for dyslipidemia, prediabetes, or hypertension; additionally, there is an association between early-onset androgenetic alopecia and Parkinson's disease resulting in decreased fertility (31). Lisinopril showed in a propensity score-matched cohort study that 52.4% of patients had died from the prescribed medication; however, it was effective in treating heart failure with reduced ejection fraction and had similar mortality rates with other prescribed medication (e.g., enalapril) (32).

Multivitamins

Supplements like multivitamins have inconclusive evidence being beneficial or harmful to cognitive outcomes, like Alzheimer-type dementia and Parkinson's disease (33, 34). Multivitamins use was commonly used for indications other than Parkinson's disease (34). Multivitamin supplements did not show as a protective factor against chronic conditions or death (35, 36).

Omega3

Improvement of cognitive function was associated with increased baseline and increment of omega-3 index (37), and non-docosahexaenoic acid omega-3 may be have positive effects (38). omega-3 polyunsaturated fatty acids show evidence of improving Parkinson's disease as well (39). Omega-3 polyunsaturated fatty acids have shown positive effects on locomotory alterations in parkinsonism (40). Circulating omega-3 polyunsaturated fatty acids showed a strong inverse association with all-cause cancer and cardiovascular disease mortality (41).

Perindopril

Perindopril has been documented to improve neurodegenerative disorders like dementia, Alzheimer's Disease, and Parkinson's disease (42). Perindopril additionally was shown to manage motor fluctuations and dyskinesia in Parkinson's disease (43). In animal models, perindopril showed protective effects against 1-methyl-4-phenyl-1,2,3,6 tetrahydropyridine-induced striatal dopamine and DOPAC depletion and mitigated severity of L-3, 4-dihydroxyphenylalanine induced dyskinesia in Parkinson's disease (44, 45). In a large study using private health insurance claims, the risk-adjusted all-cause mortality rate was lower with perindopril compared to enalapril but not losartan (common renin-angiotensin-aldosterone system inhibitors) (46).

Ramipril

Ramipril treatment has been documented to improve neurodegenerative disorders like dementia, Alzheimer's Disease, and Parkinson's disease (42). However, in one study, cognitive impairment occurred in 8% of patients allocated ramipril or given in a combination treatment, and cognitive decline was at 17% in patients allocated ramipril or given in a combination treatment (47). Despite this, ramipril does show reduced cardiovascular and cerebral risk (48), along with decreasing risk for all-cause mortality, cardiovascular mortality, and first hospitalization (32, 49).

Vitamin C

Vitamin C at increased levels of intake was associated with improved executive functioning (50). Those with Alzheimer's disease had significantly higher prevalence rates of vitamin C than those without cognitive impairments (51). Additionally, vitamin C (ascorbate) was positively associated with cognitive

function in Parkinson's disease (52). Levodopa, a common drug for somatic management of Parkinson's disease, showed improved absorption with vitamin C intake (53). However, vitamin C intake did show some associations with breast cancer and kidney stones, but the benefits of vitamin C intake (e.g., positive respiratory, neurological, ophthalmologic, musculoskeletal, renal, and dental outcomes) outweigh the potential risks (54).

Glucosamine

Habitual glucosamine supplement intake showed a lower risk of dementia, with potential causal associations refarming risk reduction (55, 56). Additionally, O-linked N-acetyl-glucosamine showed that at increased levels may slow synucleinopathies, including Parkinson's disease; but, may be detrimental to neurons and increase α-synuclein accumulation (57, 58). Habitual glucosamine supplement intake was shown to lower mortality for all causes, cancer, cardiovascular disease, respiratory and digestive diseases (59).

cod liver oil

Cod liver oil was associated with increased 25-hydroxy-vitamin D levels, and several studies suggested older adults cognitively impaired have lower 25-hydroxy-vitamin D levels (60). Cod liver oil is a common medication used for other indications other than Parkinson's disease (34). Vitamin D3, found naturally in cod liver oil, should be given to those with vitamin D deficiency, especially those with a history of falls, nontraumatic fractures, and osteoporosis (61). Additionally, coronary heart disease mortality showed as lower among omega-3 polyunsaturated fatty acids, mainly from cod liver oil (62).

primrose oil

Evening primrose oil showed improved motor performance, lowered inflammatory indicators, repaired dopamine quantities, and improved neuro histopathological lesions in rat models of Parkinson's disease (63). There have been studies that have shown evening primrose oil to be an anti-coagulant and anti-platelet with the potential to reduce cardiovascular morbidity and mortality in rat models (64). However, evening primrose oil is not well known if it is beneficial in high-risk women (65). There is currently insufficient information regarding the effects of evening primrose oil on cognition.

garlic

Garlic has been shown to have beneficial effects, such as protecting against amyloid-beta-induced neurotoxicity (66); however, at higher levels of consumption, reduced cognitive flexibility and speed processing(66), yet longitudinally such decline was not found (66). In a review of Persian medicine, garlic was found to have strong anti-Alzheimer's disease activities (67). Additionally, Persian Medicines such as garlic possess potent antioxidants and anti-inflammatory properties, target intra-plaque ferroptosis, lower lipid peroxidation and the risk of mortality, and Parkinson's disease-preventing properties (68, 69, 70).

chondroitin

Chondroitin sulfate proteoglycan-related enzymes show relationships to cognitive function. Removal of chondroitin sulfate proteoglycan negatively affects memantine on cognitive improvement in mice (71). Fucosylated chondroitin sulfate taken from sea cucumbers demonstrated positive effects in Parkinson's disease mouse models by reducing inflammation in the gut microbial dysbiosis (72). In a national sample of US adults, regular chondroitin intake was associated with reduced all-cause and cardiovascular disease mortality (73).

Supplementary Table 4. Latent classes of commonly used medications vs. transitions 2 and 3, as mediated through polypharmacy as defined by a cutoff of 5+ medications (POLYPH^{5+}): direct and indirect effects: UK Biobank 2006-2021

LC	Outcome	LC→POLYPH^{5+}			LC→Outcome			POLYPH^{5+}→Outcome		
		β	(SE)	P	β	(SE)	P	β	(SE)	P
1	Healthy to Dementia	**0.17**	**0.01**	**<0.001**	-0.02	0.04	0.64	**0.29**	**0.03**	**<0.001**
2	Healthy to Dementia	**0.81**	**0.01**	**<0.001**	-0.03	0.03	0.32	**0.29**	**0.03**	**<0.001**
3	Healthy to Dementia	**-0.38**	**0.03**	**<0.001**	+0.019	0.084	0.82	**0.29**	**0.03**	**<0.001**
4	Healthy to Dementia	**2.71**	**0.03**	**<0.001**	-0.14	0.08	0.08	**0.30**	**0.03**	**<0.001**
5	Healthy to Dementia	**0.24**	**0.02**	**<0.001**	-0.01	0.06	0.86	**0.29**	**0.03**	**<0.001**
6	Healthy to Dementia	**-3.00**	**0.02**	**<0.001**	-0.02	0.03	0.63	**0.28**	**0.03**	**<0.001**
7	Healthy to Dementia	**1.42**	**0.02**	**<0.001**	**-0.13**	**0.05**	**0.01**	**0.30**	**0.03**	**<0.001**
8	Healthy to Dementia	**0.76**	**0.08**	**<0.001**	0.08	0.21	0.70	**0.29**	**0.03**	**<0.001**
9	Healthy to Dementia	**1.21**	**0.02**	**<0.001**	**0.23**	**0.04**	**<0.001**	**0.26**	**0.03**	**<0.001**
1	Healthy to Mortality	**0.17**	**0.01**	**<0.001**	0.03	0.02	0.07	**0.27**	**0.01**	**<0.001**
2	Healthy to Mortality	**0.81**	**0.01**	**<0.001**	**-0.04**	**0.02**	**0.02**	**0.27**	**0.01**	**<0.001**

**3**	**Healthy to Mortality**	**-0.381**	**0.028**	**<0.001**	**-0.114**	**0.038**	**0.002**	**0.26**	**0.01**	**<0.001**
**4**	**Healthy to Mortality**	**2.71**	**0.03**	**<0.001**	**-0.22**	**0.04**	**<0.001**	**0.28**	**0.02**	**<0.001**
**5**	**Healthy to Mortality**	**0.24**	**0.02**	**<0.001**	**0.06**	**0.03**	**0.03**	**0.27**	**0.01**	**<0.001**
**6**	**Healthy to Mortality**	**-3.00**	**0.02**	**<0.001**	**0.08**	**0.01**	**<0.001**	**0.30**	**0.02**	**<0.001**
**7**	**Healthy to Mortality**	**1.42**	**0.02**	**<0.001**	**-0.21**	**0.02**	**<0.001**	**0.28**	**0.01**	**<0.001**
**8**	**Healthy to Mortality**	**0.76**	**0.08**	**<0.001**	**0.40**	**0.09**	**<0.001**	**0.26**	**0.01**	**<0.001**
**9**	**Healthy to Mortality**	**1.21**	**0.02**	**<0.001**	**0.07**	**0.02**	**<0.001**	**0.26**	**0.02**	**<0.001**

Abbreviations:

LC=Latent class; P=P-value; POLYPH^{5+}=Polypharmacy with 5+ medications cutoff; SE = Standard error; UK=United Kingdom.

Supplementary Figure 1. Participant Flowchart

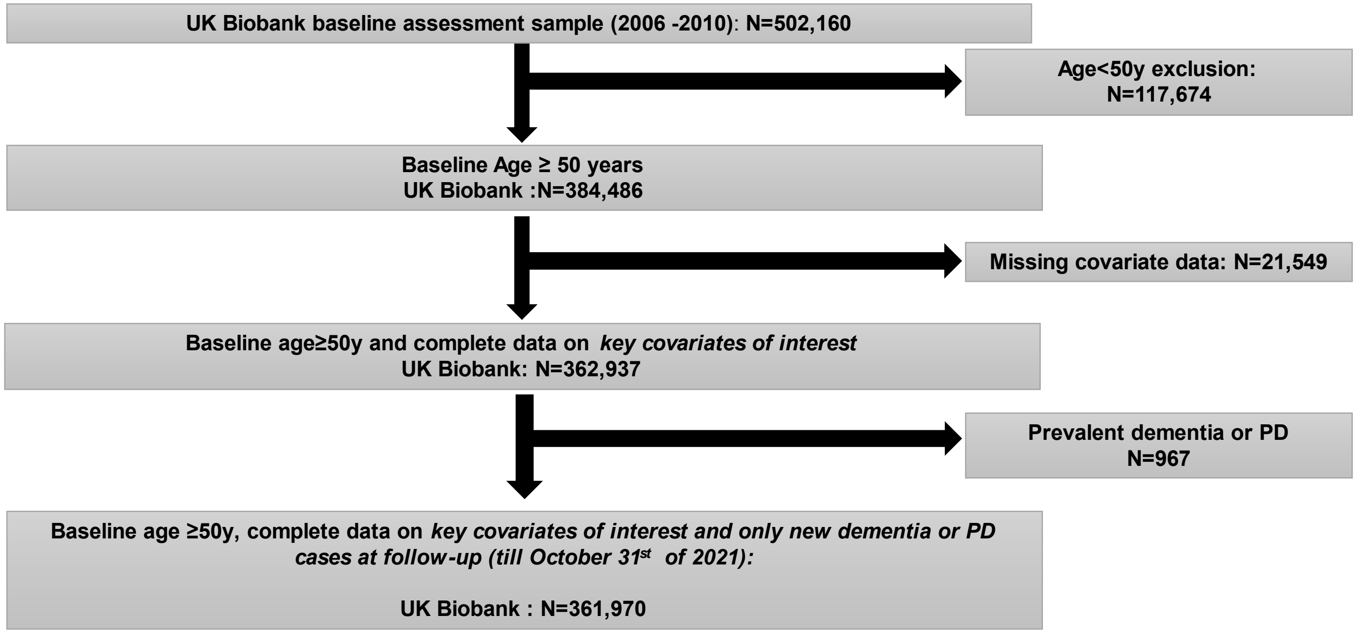

FIGURE S2. Probabilities of being in each of 4 states: UK Biobank 2006 -2021

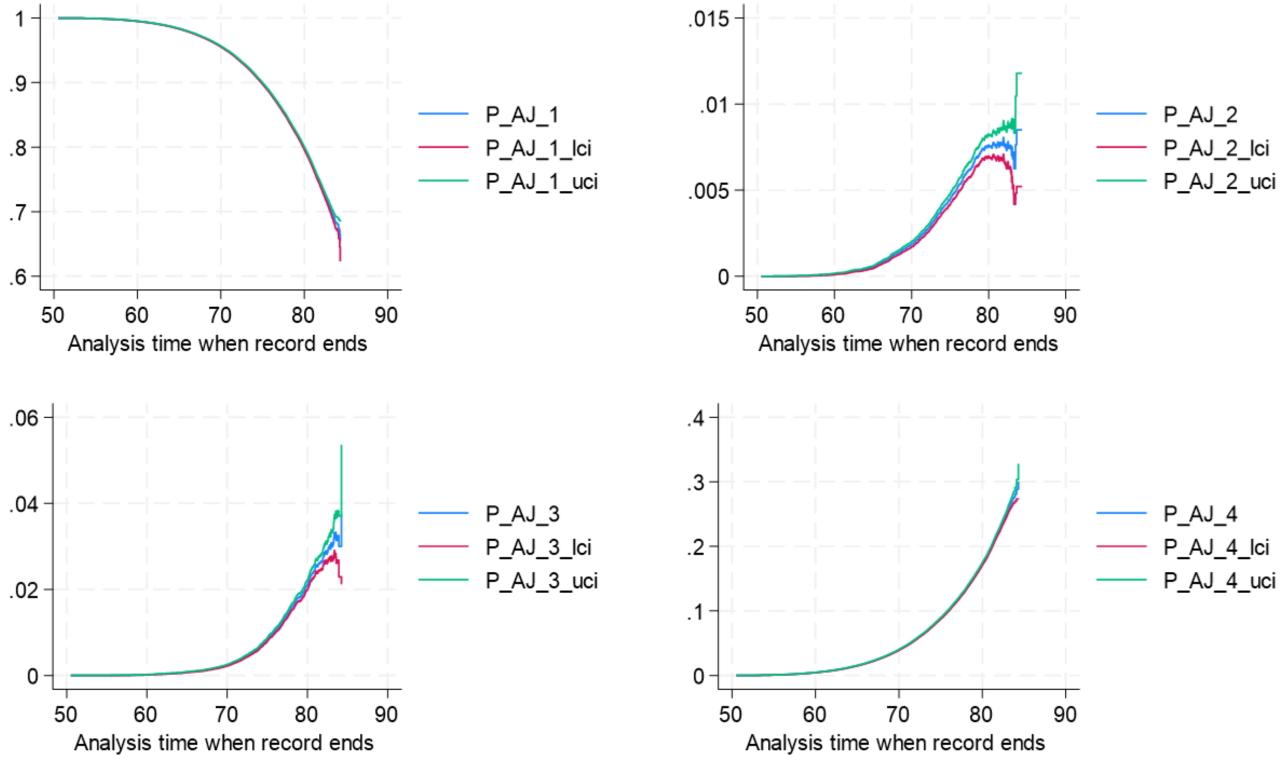

State 1: Healthy, State 2: PD, State 3: Dementia, State 4: Death

FIGURE S3. Transition probabilities from PD and Dementia across follow -up age: UK Biobank 2006-2021

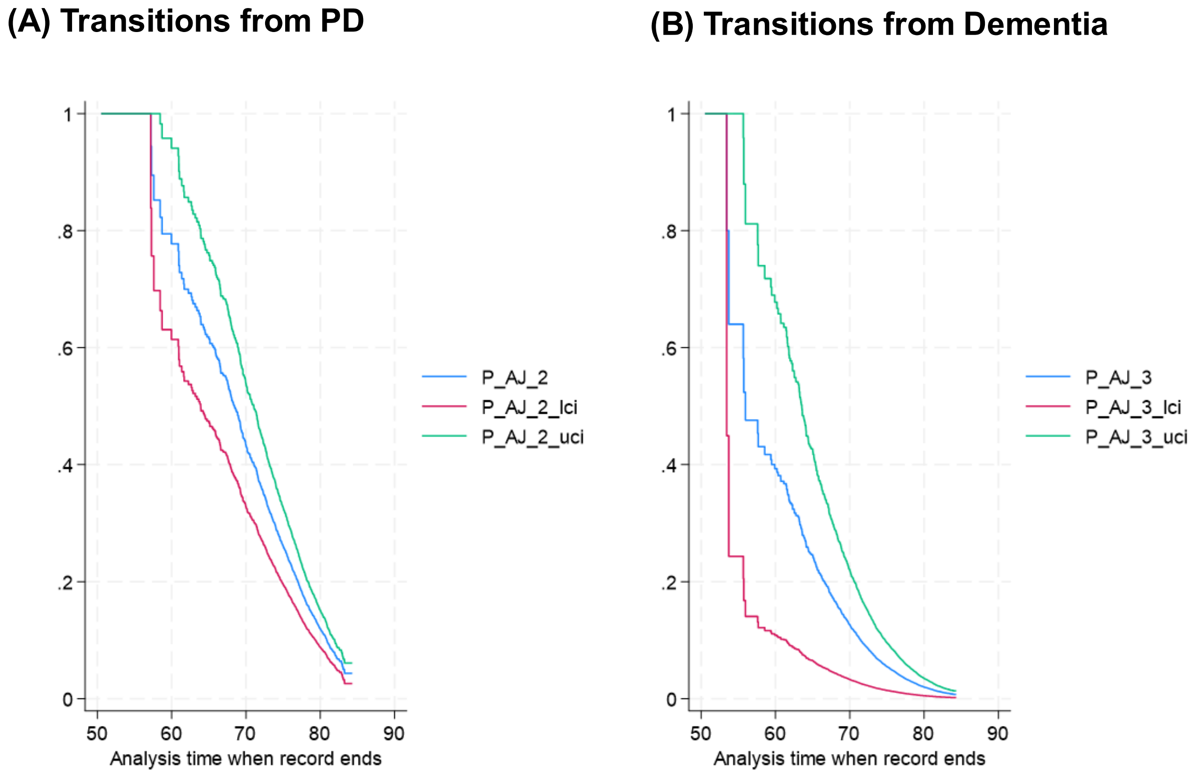

REFERENCES

1. Putter H, Fiocco M, Geskus RB. Tutorial in biostatistics: competing risks and multi-state models. Stat Med. 2007;26(11):2389-430.
2. Crowther MJ. merlin—A unified modeling framework for data analysis and methods development in Stata. The Stata Journal. 2020;20(4):763-84.
3. Royston P, Parmar MK. Flexible parametric proportional-hazards and proportional-odds models for censored survival data, with application to prognostic modelling and estimation of treatment effects. Stat Med. 2002;21(15):2175-97.
4. Gutierrez RG, Linhart, J. M., Pitblado, J. S.,. From the help desk: Local polynomial regression and Stata plugins. Stata Journal. 2003;3:412-9.
5. Schreiber JB. Latent Class Analysis: An example for reporting results. Res Social Adm Pharm. 2017;13(6):1196-201.
6. McCutcheon A. Latent Class Analysis. Sage Publications1987.
7. Collins LM, Lanza, S. T.,. Latent Class and Latent Transition Analysis With Applications in the Social, Behavioral, and Health Sciences: Wiley; 2013.
8. Rabe-Hesketh S. SA. Multilevel and Longitudinal Modeling Using Stata, Volume II: Categorical Responses, Counts, and Survival, Third Edition. 3rd Edition2008.
9. Skrondal A, Rabe-Hesketh, S., . Generalized Latent Variable Modeling: Multilevel, Longitudinal, and Structural Equation Models: Chapman & Hall/CRC; 2004.
10. Joost A, Schunkert H, Radke PW. Candesartan cilexetil: an update. Expert Opin Pharmacother. 2011;12(11):1769-80.
11. Wu L, Tian YY, Shi JP, Xie W, Shi JQ, Lu J, et al. Inhibition of endoplasmic reticulum stress is involved in the neuroprotective effects of candesartan cilexitil in the rotenone rat model of Parkinson's disease. Neurosci Lett. 2013;548:50-5.
12. Erdmann E, George M, Voet B, Belcher G, Kolb D, Hiemstra S, et al. The safety and tolerability of candesartan cilexetil in CHF. J Renin Angiotensin Aldosterone Syst. 2000;1 Suppl 1:31-6.
13. Baguet JP, Barone-Rochette G, Neuder Y. Candesartan cilexetil in the treatment of chronic heart failure. Vasc Health Risk Manag. 2009;5(1):257-64.
14. Gomes CM, Sammour ZM, Bessa Junior Jd, Barbosa ER, Lopes RI, Sallem FS, et al. Neurological status predicts response to alpha-blockers in men with voiding dysfunction and Parkinson's disease. Clinics. 2014;69:817-22.
15. Wucherer D, Eichler T, Hertel J, Kilimann I, Richter S, Michalowsky B, et al. Potentially inappropriate medication in community-dwelling primary care patients who were screened positive for dementia. Journal of Alzheimer's Disease. 2017;55(2):691-701.
16. Hart A, Aldridge G, Zhang Q, Narayanan NS, Simmering JE. Association of Terazosin, Doxazosin, or Alfuzosin Use and Risk of Dementia With Lewy Bodies in Men. Neurology. 2024;103(2):e209570.
17. Lamichhane P, Tariq A, Akhtar AN, Raza M, Lamsal AB, Agrawal A. Risk of Parkinson's disease among users of alpha-adrenergic receptor antagonists: a systematic review and meta-analysis. Annals of Medicine and Surgery. 2024;86(6):3409-15.
18. Weber MA, Sivakumar K, Tabakovic EE, Oya M, Aldridge GM, Zhang Q, et al. Glycolysis-enhancing α1-adrenergic antagonists modify cognitive symptoms related to Parkinson's disease. npj Parkinson's Disease. 2023;9(1):32.
19. Rose L, Graham L, Koenecke A, Powell M, Xiong R, Shen Z, et al. The association between alpha-1 adrenergic receptor antagonists and in-hospital mortality from COVID-19. Frontiers in Medicine. 2021;8:637647.
20. Rhea EM, Leclerc M, Yassine HN, Capuano AW, Tong H, Petyuk VA, et al. State of the Science on Brain Insulin Resistance and Cognitive Decline Due to Alzheimer's Disease. Aging Dis. 2024;15(4):1688-725.

21. Abdalla MMI. Insulin resistance as the molecular link between diabetes and Alzheimer's disease. World J Diabetes. 2024;15(7):1430-47.
22. Bai W, An S, Jia H, Xu J, Qin L. Relationship between triglyceride-glucose index and cognitive function among community-dwelling older adults: a population-based cohort study. Front Endocrinol (Lausanne). 2024;15:1398235.
23. Mesarosova L, Scheper M, Iyer A, Anink JJ, Mills JD, Aronica E. miR-193b-3p/ PGC-1alpha pathway regulates an insulin dependent anti-inflammatory response in Parkinson's disease. Neurobiol Dis. 2024;199:106587.
24. Fan J, Aihemaiti A, Yidilisi A, Liu X, Wang J. Association between triglyceride-glucose index and all-cause mortality in patients underwent transcatheter aortic valve replacement. BMC Cardiovasc Disord. 2024;24(1):508.
25. Xing Z, Schocken DD, Zgibor JC, Alman AC. Course and trajectories of insulin resistance, incident heart failure and all-cause mortality in nondiabetic people. Endocrine. 2024.
26. Imtiaz A, Shimonaka S, Uddin MN, Elahi M, Ishiguro K, Hasegawa M, et al. Selection of lansoprazole from an FDA-approved drug library to inhibit the Alzheimer's disease seed-dependent formation of tau aggregates. Front Aging Neurosci. 2024;16:1368291.
27. Kramer V, Brooks AF, Haeger A, Kuljis RO, Rafique W, Koeppe RA, et al. Evaluation of [(18)F]-N-Methyl lansoprazole as a Tau PET Imaging Agent in First-in-Human Studies. ACS Chem Neurosci. 2020;11(3):427-35.
28. Howden CW. Management of acid-related disorders in patients with dysphagia. Am J Med. 2004;117 Suppl 5A:44S-8S.
29. Sobek J, Li J, Combes BF, Gerez JA, Henrich MT, Geibl FF, et al. Efficient characterization of multiple binding sites of small molecule imaging ligands on amyloid-beta, tau and alpha-synuclein. Eur J Nucl Med Mol Imaging. 2024.
30. Du XL, Simpson LM, Osani MC, Yama JM, Davis BR. Risk of Developing Alzheimer's Disease and Related Dementias in ALLHAT Trial Participants Receiving Diuretic, ACE-Inhibitor, or Calcium-Channel Blocker with 18 Years of Follow-Up. J Alzheimers Dis Parkinsonism. 2022;12(3).
31. Liu LP, Wariboko MA, Hu X, Wang ZH, Wu Q, Li YM. Factors associated with early-onset androgenetic alopecia: A scoping review. PLoS One. 2024;19(3):e0299212.
32. Frohlich H, Henning F, Tager T, Schellberg D, Grundtvig M, Goode K, et al. Comparative effectiveness of enalapril, lisinopril, and ramipril in the treatment of patients with chronic heart failure: a propensity score-matched cohort study. Eur Heart J Cardiovasc Pharmacother. 2018;4(2):82-92.
33. Butler M, Nelson VA, Davila H, Ratner E, Fink HA, Hemmy LS, et al. Over-the-Counter Supplement Interventions to Prevent Cognitive Decline, Mild Cognitive Impairment, and Clinical Alzheimer-Type Dementia: A Systematic Review. Ann Intern Med. 2018;168(1):52-62.
34. Ferry P, Johnson M, Wallis P. Use of complementary therapies and non-prescribed medication in patients with Parkinson's disease. Postgrad Med J. 2002;78(924):612-4.
35. Sesso HD, Rist PM, Aragaki AK, Rautiainen S, Johnson LG, Friedenberg G, et al. Multivitamins in the prevention of cancer and cardiovascular disease: the COcoa Supplement and Multivitamin Outcomes Study (COSMOS) randomized clinical trial. Am J Clin Nutr. 2022;115(6):1501-10.
36. O'Connor EA, Evans CV, Ivlev I, Rushkin MC, Thomas RG, Martin A, et al. Vitamin and Mineral Supplements for the Primary Prevention of Cardiovascular Disease and Cancer: Updated Evidence Report and Systematic Review for the US Preventive Services Task Force. JAMA. 2022;327(23):2334-47.
37. He X, Yu H, Fang J, Qi Z, Pei S, Yan B, et al. The effect of n-3 polyunsaturated fatty acid supplementation on cognitive function outcomes in the elderly depends on the baseline omega-3 index. Food Funct. 2023;14(21):9506-17.
38. Sala-Vila A, Tintle N, Westra J, Harris WS. Plasma Omega-3 Fatty Acids and Risk for Incident Dementia in the UK Biobank Study: A Closer Look. Nutrients. 2023;15(23).
39. Li P, Song C. Potential treatment of Parkinson's disease with omega-3 polyunsaturated fatty acids. Nutr Neurosci. 2022;25(1):180-91.

40. Barroso-Hernandez A, Ramirez-Higuera A, Pena-Montes C, Cortes-Ramirez SA, Rodriguez-Dorantes M, Lopez-Franco O, et al. Beneficial effects of an algal oil rich in omega-3 polyunsaturated fatty acids on locomotor function and D(2) dopamine receptor in haloperidol-induced parkinsonism. Nutr Neurosci. 2022;25(3):519-29.
41. Zhang Y, Sun Y, Yu Q, Song S, Brenna JT, Shen Y, et al. Higher ratio of plasma omega-6/omega-3 fatty acids is associated with greater risk of all-cause, cancer, and cardiovascular mortality: A population-based cohort study in UK Biobank. Elife. 2024;12.
42. Kaur P, Muthuraman A, Kaur M. The implications of angiotensin-converting enzymes and their modulators in neurodegenerative disorders: current and future perspectives. ACS Chem Neurosci. 2015;6(4):508-21.
43. Reardon KA, Mendelsohn FA, Chai SY, Horne MK. The angiotensin converting enzyme (ACE) inhibitor, perindopril, modifies the clinical features of Parkinson's disease. Aust N Z J Med. 2000;30(1):48-53.
44. Kurosaki R, Muramatsu Y, Imai Y, Kato H, Araki T. Neuroprotective effect of the angiotensin-converting enzyme inhibitor perindopril in MPTP-treated mice. Neurol Res. 2004;26(6):644-57.
45. Park HY, Lee GS, Go J, Ryu YK, Lee CH, Moon JH, et al. Angiotensin-converting enzyme inhibition prevents l-dopa-induced dyskinesia in a 6-ohda-induced mouse model of Parkinson's disease. Eur J Pharmacol. 2024;973:176573.
46. Snyman JR, Gumedze F, Jones ESW, Alaba OA, Tsabedze N, Vira A, et al. Comparing Cardiovascular Outcomes and Costs of Perindopril-, Enalapril- or Losartan-Based Antihypertensive Regimens in South Africa: Real-World Medical Claims Database Analysis. Adv Ther. 2023;40(11):5076-89.
47. Anderson C, Teo K, Gao P, Arima H, Dans A, Unger T, et al. Renin-angiotensin system blockade and cognitive function in patients at high risk of cardiovascular disease: analysis of data from the ONTARGET and TRANSCEND studies. Lancet Neurol. 2011;10(1):43-53.
48. Zimmermann M, Unger T. Challenges in improving prognosis and therapy: the Ongoing Telmisartan Alone and in Combination with Ramipril Global End point Trial programme. Expert Opin Pharmacother. 2004;5(5):1201-8.
49. Hsing SC, Lu KC, Sun CA, Chien WC, Chung CH, Kao SY. The Association of Losartan and Ramipril Therapy With Kidney and Cardiovascular Outcomes in Patients With Chronic Kidney Disease: A Chinese Nation-Wide Cohort Study in Taiwan. Medicine (Baltimore). 2015;94(48):e1999.
50. White SA, Ward N, Verghese J, Kramer AF, Grandjean da Costa K, Liu CK, et al. Nutritional Risk Status, Dietary Intake and Cognitive Performance in Older Adults with Motoric Cognitive Risk Syndrome. JAR Life. 2020;9:47-54.
51. Lanyau-Dominguez Y, Macias-Matos C, Jesus J, Maria G, Suarez-Medina R, Eugenia M, et al. Levels of Vitamins and Homocysteine in Older Adults with Alzheimer Disease or Mild Cognitive Impairment in Cuba. MEDICC Rev. 2020;22(4):40-7.
52. Spencer ES, Pitcher T, Veron G, Hannam T, MacAskill M, Anderson T, et al. Positive Association of Ascorbate and Inverse Association of Urate with Cognitive Function in People with Parkinson's Disease. Antioxidants (Basel). 2020;9(10).
53. Boelens Keun JT, Arnoldussen IA, Vriend C, van de Rest O. Dietary Approaches to Improve Efficacy and Control Side Effects of Levodopa Therapy in Parkinson's Disease: A Systematic Review. Adv Nutr. 2021;12(6):2265-87.
54. Xu K, Peng R, Zou Y, Jiang X, Sun Q, Song C. Vitamin C intake and multiple health outcomes: an umbrella review of systematic reviews and meta-analyses. Int J Food Sci Nutr. 2022;73(5):588-99.
55. Xu C, Hou Y, Fang X, Yang H, Cao Z. The role of type 2 diabetes in the association between habitual glucosamine use and dementia: a prospective cohort study. Alzheimers Res Ther. 2022;14(1):184.
56. Zheng J, Ni C, Zhang Y, Huang J, Hukportie DN, Liang B, et al. Association of regular glucosamine use with incident dementia: evidence from a longitudinal cohort and Mendelian randomization study. BMC Med. 2023;21(1):114.

57. Marotta NP, Lin YH, Lewis YE, Ambroso MR, Zaro BW, Roth MT, et al. O-GlcNAc modification blocks the aggregation and toxicity of the protein alpha-synuclein associated with Parkinson's disease. Nat Chem. 2015;7(11):913-20.
58. Wani WY, Ouyang X, Benavides GA, Redmann M, Cofield SS, Shacka JJ, et al. O-GlcNAc regulation of autophagy and alpha-synuclein homeostasis; implications for Parkinson's disease. Mol Brain. 2017;10(1):32.
59. Li ZH, Gao X, Chung VC, Zhong WF, Fu Q, Lv YB, et al. Associations of regular glucosamine use with all-cause and cause-specific mortality: a large prospective cohort study. Ann Rheum Dis. 2020;79(6):829-36.
60. Eymundsdottir H, Chang M, Geirsdottir OG, Gudmundsson LS, Jonsson PV, Gudnason V, et al. Lifestyle and 25-hydroxy-vitamin D among community-dwelling old adults with dementia, mild cognitive impairment, or normal cognitive function. Aging Clin Exp Res. 2020;32(12):2649-56.
61. Charoenngam N, Shirvani A, Holick MF. Vitamin D for skeletal and non-skeletal health: What we should know. J Clin Orthop Trauma. 2019;10(6):1082-93.
62. Lentjes MAH, Keogh RH, Welch AA, Mulligan AA, Luben RN, Wareham NJ, et al. Longitudinal associations between marine omega-3 supplement users and coronary heart disease in a UK population-based cohort. BMJ Open. 2017;7(10):e017471.
63. Mohammad HMF, El-Baz AA, Mahmoud OM, Khalil S, Atta R, Imbaby S. Protective effects of evening primrose oil on behavioral activities, nigral microglia and histopathological changes in a rat model of rotenone-induced parkinsonism. J Chem Neuroanat. 2023;127:102206.
64. Riaz A, Khan RA, Ahmed SP. Assessment of anticoagulant effect of evening primrose oil. Pak J Pharm Sci. 2009;22(4):355-9.
65. Duley L. Pre-eclampsia, eclampsia, and hypertension. BMJ Clin Evid. 2011;2011.
66. Dominguez LJ, Barbagallo M. Nutritional prevention of cognitive decline and dementia. Acta Biomed. 2018;89(2):276-90.
67. Iranshahy M, Javadi B. Diet therapy for the treatment of Alzheimer's disease in view of traditional Persian medicine: A review. Iran J Basic Med Sci. 2019;22(10):1102-17.
68. Iranshahy M, Javadi B, Sahebkar A. Protective effects of functional foods against Parkinson's disease: A narrative review on pharmacology, phytochemistry, and molecular mechanisms. Phytother Res. 2022;36(5):1952-89.
69. Gao T, Gao S, Wang H, Wang S, Li L, Hu J, et al. Garlic ameliorates atherosclerosis by regulating ferroptosis pathway: an integrated strategy of network pharmacology, bioinformatic and experimental verification. Front Pharmacol. 2024;15:1388540.
70. Jones WO, Symons LE. Protein synthesis in the whole body, liver, skeletal muscle and kidney cortex of lambs infected by the nematode Trichostrongylus colubriformis. Int J Parasitol. 1982;12(4):295-301.
71. Maeda S, Yamada J, Iinuma KM, Nadanaka S, Kitagawa H, Jinno S. Chondroitin sulfate proteoglycan is a potential target of memantine to improve cognitive function via the promotion of adult neurogenesis. Br J Pharmacol. 2022;179(20):4857-77.
72. Liu Y, Liu X, Ye Q, Wang Y, Zhang J, Lin S, et al. Fucosylated Chondroitin Sulfate against Parkinson's Disease through Inhibiting Inflammation Induced by Gut Dysbiosis. J Agric Food Chem. 2022;70(42):13676-91.
73. King DE, Xiang J. Glucosamine/Chondroitin and Mortality in a US NHANES Cohort. J Am Board Fam Med. 2020;33(6):842-7.
